# Generalized Machine Learning Potentials for Predicting Low-Pressure Water Adsorption in Flexible Al-Based Metal–Organic Frameworks

**DOI:** 10.1021/acs.jctc.6c01162

**Published:** 2026-08-25

**Authors:** Yutao Li, Xiaoqi Zhang, Xin Jin, Berend Smit

**Affiliations:** Laboratory of Molecular Simulation (LSMO), Institut des Sciences et Ingénierie Chimiques, 27218 École Polytechnique Fédérale de Lausanne (EPFL), Rue de l’Industrie 17, CH-1951 Sion, Switzerland

## Abstract

Metal–organic frameworks (MOFs) are promising materials for adsorption and separation, but accurately predicting water adsorption remains a major challenge in molecular simulation. Classical force fields, such as UFF, often fail to capture the strong, directional hydrogen-bonding interactions between water and MOFs, while high-throughput density functional theory (DFT) calculations are computationally prohibitive. Here, we develop a transferable machine-learning potential (MLP) for water adsorption in Al-based MOFs by fine-tuning the pretrained MACE foundation model. Using a data set of 420 Al-MOFs with diverse chemical environments, we show that accurate prediction of water adsorption thermodynamics requires not only an improved description of water–framework interactions but also explicit treatment of framework flexibility. The resulting model reproduces experimental heats of adsorption and Henry coefficients for a series of benchmark Al-MOFs and achieves DFT-level accuracy across more than 400 Al-MOFs. Analysis of MIL-160 establishes practical accuracy requirements of approximately 10 kJ mol^–1^ in total energies and 40 meV Å^–1^ in atomic forces for reliable adsorption thermodynamics. Compared with DFT-level predictions, UFF underestimates water adsorption enthalpies by more than 10 kJ mol^–1^ for 74% of the materials studied. In addition, the MLP identifies lower-energy framework configurations than previously reported DFT-optimized structures, highlighting the importance of enhanced configurational sampling. These results demonstrate that foundation-model-based machine-learning potentials can enable DFT-accurate, high-throughput screening of water adsorption in flexible MOFs.

## Graphical abstract

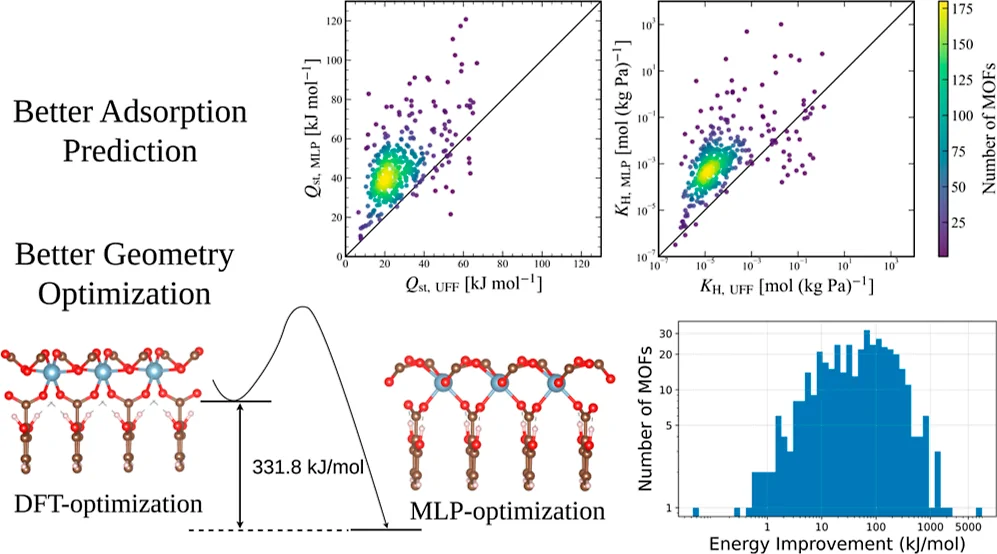

## 1 Introduction

Metal–organic frameworks (MOFs) are a promising class of porous materials for gas adsorption and separation, owing to their large capacity, tunable pore structures, and chemical versatility.^1^ To date, over 90,000 MOFs have been experimentally synthesized,^2^ and more than a trillion hypothetical structures can be generated through computational design.^3^ High-throughput screening studies^4–6^ have been widely used to identify an optimal MOF for a specific application.

Given the ubiquity of water in the environment, predicting water adsorption within MOFs is often critical for many applications. For example, water-based applications such as desiccation or atmospheric water harvesting rely on knowledge of the water uptake. In addition, in gas separations, water often competes with other target molecules, such as CO_2_, in carbon capture processes.^7,8^

Most large-scale computational screening studies are based on molecular simulations, which use generalized force fields for MOFs, such as the universal force field (UFF)^9^ or Dreiding,^10^ to predict adsorption thermodynamics. These universal force fields give reasonable predictions of the CO_2_ isotherms in most MOFs (for some exceptions, see Li et al.^11^). However, for water, these universal force fields often fail to accurately describe adsorption behavior. A signature of these difficulties can already be seen if we compare the predicted binding energy of water as predicted from a DFT calculation with the binding energy predicted from the UFF force field for a selection of 36 MOFs (see Figure 1). Only for a very small number of MOFs, the UFF gives a reasonable description of the water–MOF interactions. There are several reasons why we see such a large difference. In the force field calculations, we assume that charges of both MOFs and H_2_O are fixed. Hence, polarization effects are not considered.^12^ In addition, for water, a correct description of the hydrogen bond network is essential; a classical force field and a rigid MOF impose constraints that can impact the accuracy with which these models predict adsorption properties.^4,13,14^ One can expect that these effects will also affect the formation of hydrogen bonds, making H_2_O particularly problematic to describe with these classical force fields.

**Figure 1:**
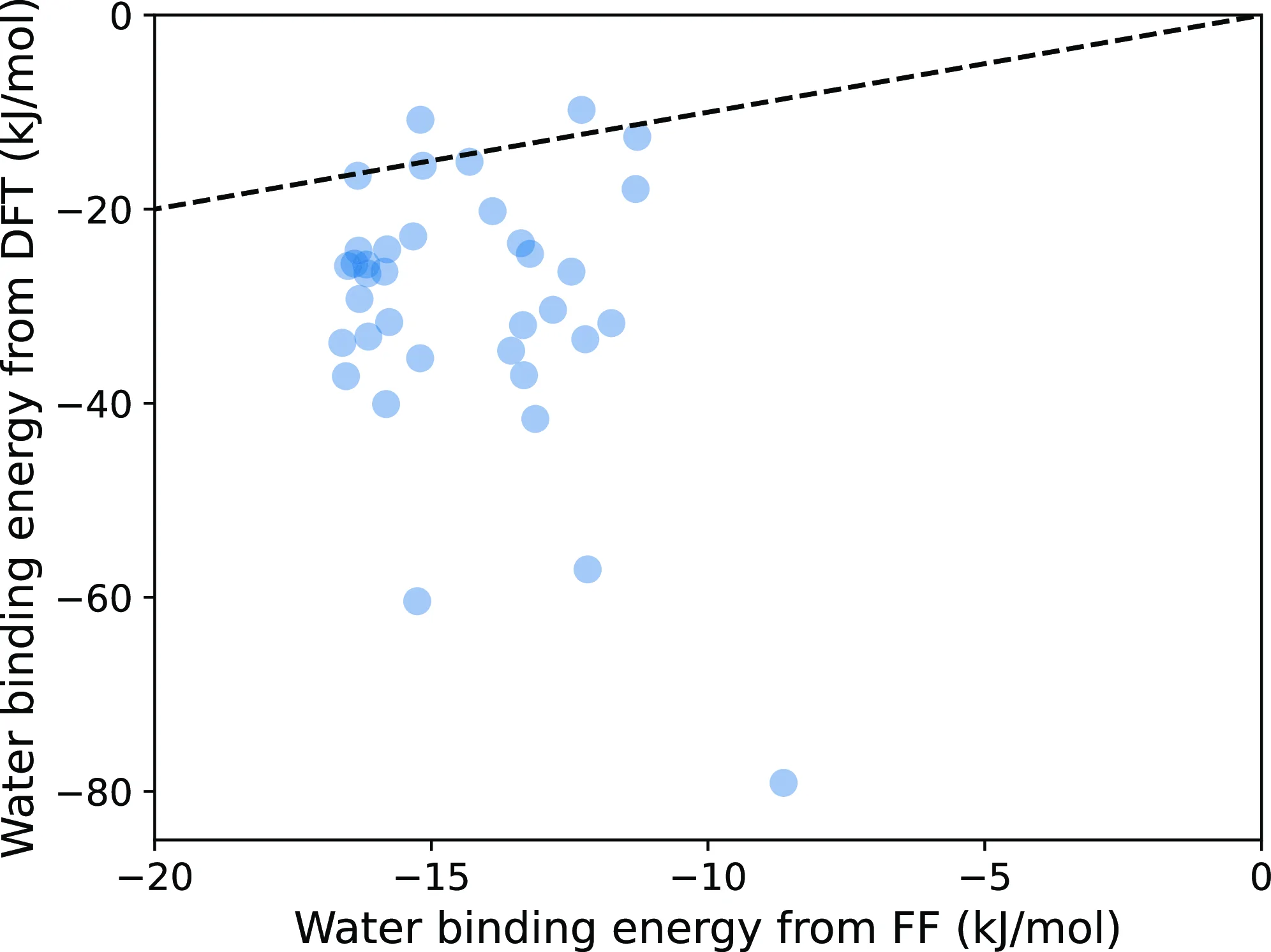
Comparison of water binding energies for a structurally diverse subset of 36 MOFs (selected from the PrISMa data set^15^) from DFT and the binding energies from UFF. Starting from DFT-optimized MOF structures, UFF was used to search the water-binding sites and calculate binding energies. These minimum-energy positions of H_2_O from DFT were used as a starting point for the optimization with DFT. In both cases, the MOF was kept rigid.

To better describe the water–MOF interactions, Goeminne and Van Speybroeck^16^ trained a machine learning potential (MLP) and successfully reproduced the experimental water isotherms for MOF-303, MOF-333, and MOF-LA2-1. Furthermore, they showed that ignoring framework flexibility leads to an underestimation of water isotherms for MOF-303, MOF-333, and MOF-LA2-1. Goeminne and Van Speybroeck^16^ trained their MLP from scratch, a process that requires a large number of DFT calculations. An alternative is to fine-tune a foundation model (e.g., MACE,^17^ EquiformerV2,^18^ orb-v3,^19^ DPA-1 ^20^), which could give us the same accuracy with a smaller number of DFT calculations, as we can leverage the knowledge contained in the training of the foundational model.

For example, Lim et al.^21^ fine-tuned the MACE-MP-0b2 foundation model to improve the description of the H_2_O–MOFs and CO_2_–MOFs interactions. They used the fine-tuned model to calculate zero-loading heats of adsorption of water (*Q*_st_(H_2_O)) and CO_2_ (*Q*_st_(CO_2_)). Water adsorption properties in the zero-loading limit (e.g., adsorption enthalpy or Henry coefficient) are used as a key performance indicator (KPI) in high-throughput screening studies.^4,13^ The work of Lim et al.^21^ shows a significant improvement over the MACE model’s predictions without fine-tuning. However, their predictions have an average error of about 20 kJ mol^–1^ per configuration in the heat of adsorption. Lim et al.^21^ assumed the framework was rigid and fine-tuned the MACE model using DFT calculations of 6 configurations near the binding site for 60 MOFs, for both H_2_O and CO_2_. Typical heats of adsorptions in MOF are 10 kJ mol^–1^ to 60 kJ mol^–1^. An average error of 20 kJ mol^–1^ per configuration, therefore, results in an uncertainty that is too large for most practical applications. This motivated the current work, in which we fine-tune the MACE-MP-0b2 foundation model for a set of Al-containing MOFs to achieve more accurate predictions of the heat of adsorption of water. To achieve this accuracy, we allowed the framework to be flexible and developed a strategy to optimize the number of configurations for which we need to perform DFT calculations.

We show that for those Al-MOFs, for which accurate experimental data is available (CAU-10-X^22^ (X = H, NH_2_ and NO_2_), MIP-211,^35^ MFM-300,^23^ KMF-1,^24^ MIL-160 ^25–27^ ), our predicted heats of adsorption and Henry coefficient agree very well with these experimental data. In addition, we show that with only a few additional DFT calculations, our model can be extended to a much larger set of Al-MOFs.

## 2 Water Adsorption in MIL-160

To demonstrate the potential of machine learning potentials (MLPs) over the UFF force fields, we first investigate the water adsorption within MIL-160 in detail. MIL-160 is used for water-based applications, such as atmospheric water harvesting,^25^ heat reallocation,^28^ and indoor moisture control.^27^

MIL-160 consists of helical chains of corner-sharing AlO_6_ octahedra, where each Al center is coordinated by four carboxylate oxygen atoms from organic ligands and two cis-oriented hydroxyl groups (see Figure 2). These hydroxyls bridge adjacent Al centers, forming one-dimensional chains along the *c*-axis. These infinite chains are the most common type of metal nodes in our Al-MOFs data set. The organic ligands connect these chains into a 3D framework with square-shaped channels of approximately 5 Å in diameter.^28^ The experimental *Q*_st_(H_2_O) of MIL-160, reported in three independent studies,^25–27^ ranges from 49 kJ mol^–1^ to 54 kJ mol^–1^ and exhibits minimal variation across a water loading range of 0.02 g g^–1^ to 0.3 g g^–1^, making it a reliable reference for validating our simulation results.

**Figure 2:**
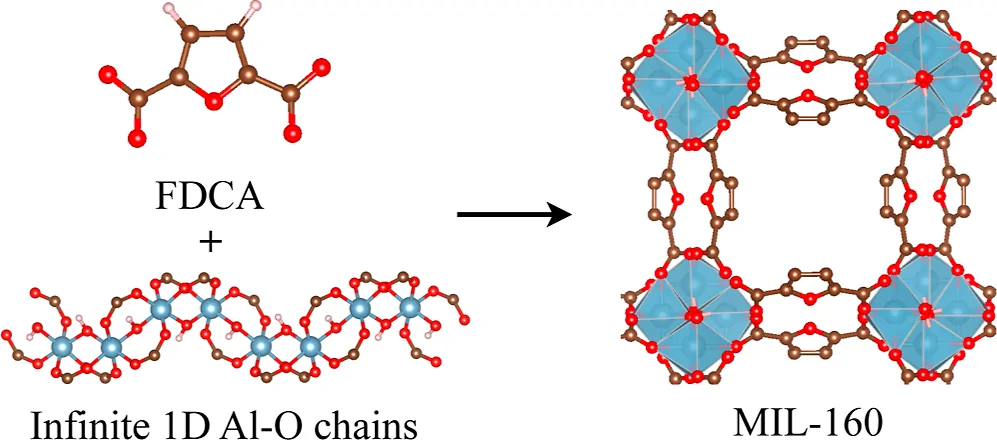
Crystal structure of MIL-160 (right) and its building blocks (left) are shown. All hydrogen atoms are omitted for simplicity, except those in the μ_2_-OH groups. In the structural diagrams, blue spheres represent aluminum atoms, red spheres represent oxygen atoms, brown spheres represent carbon atoms, and white spheres represent hydrogen atoms.

### 2.1 Heat of Adsorption

First, we use the UFF force field to calculate the heat of adsorption (*Q*_st_(H_2_O)) in the limit of zero loading, assuming the framework is rigid. The calculation yields a heat of adsorption of 33.9 kJ mol^–1^, which is significantly lower than the experimental values. The fine-tuned MACE model gave us a value of 53.3 kJ mol^–1^, consistent with the experimental results.

The MLP provides a more accurate description of the interactions between the MOF and H_2_O, particularly the hydrogen bonding between H_2_O and the μ_2_-OH group on the AlO_6_ cluster. In addition, the simulation based on the MLP can explicitly account for framework flexibility. In this case, the rotation of the μ_2_-OH group is important because its hydrogen bonds with H_2_O are sensitive to the orientation of the μ_2_-OH group. In classical simulations that assume a rigid framework, such subtle effects cannot be captured.

### 2.2 Fine-Tuning the Machine Learning Potential

The reliability of *Q*_st_(H_2_O) depends on the accuracy of the MLP, which can be evaluated on a comprehensive DFT-labeled test data set. For a single MOF, the number of DFT calculations is not a limiting factor, and it is straightforward to achieve a sufficiently accurate fine-tuned MACE model. However, to fine-tune over 400 Al-MOFs, one has to be more careful, as the total number of DFT calculations will become a bottleneck. To optimize the number of DFT calculations, we first need to establish an accuracy threshold. It is important to note that this threshold depends on the problem we want to solve. We have used our results for MIL-160 to obtain more general insights into the factors important for fine-tuning.

#### 2.2.1 Heat of Adsorption

To compute the heat of adsorption, we need to take the difference between the energies of two different systems: the MOF with H_2_O and the pristine MOF and a water molecule in the gas phase

1 $$Q_{st}=\left[\left\langle E_{MOF}\right\rangle +\left\langle E_{H_{2}O}\right\rangle \right]-\left\langle E_{MOF+H_{2}O}\right\rangle +RT$$

where $\left\langle E_{MOF+H_{2}O}\right\rangle$ , ⟨*E*_MOF_⟩, and $\left\langle E_{H_{2}O}\right\rangle$ are the ensemble averages of the energy of the MOF with a water molecule, the pristine MOF, and a water molecule in the ideal gas phase, respectively. Throughout this work, *Q*_st_ is reported as the positive magnitude of the heat of adsorption.

In Figure 3, we show the heat of adsorption for MIL-160 as a function of the number of training data points that we used for the fine-tuning. If we take MACE out of the box (zero training), we obtain a heat of adsorption of 90 kJ mol^–1^, which is about twice the experimental value of 52 kJ mol^–1^, and deviates even more than the result from the UFF classical force field (33.9 kJ mol^–1^). Figure 4 shows that with a little over 50 DFT calculations, we obtain a model that predicts the heat of adsorption with sufficient accuracy.

**Figure 3:**
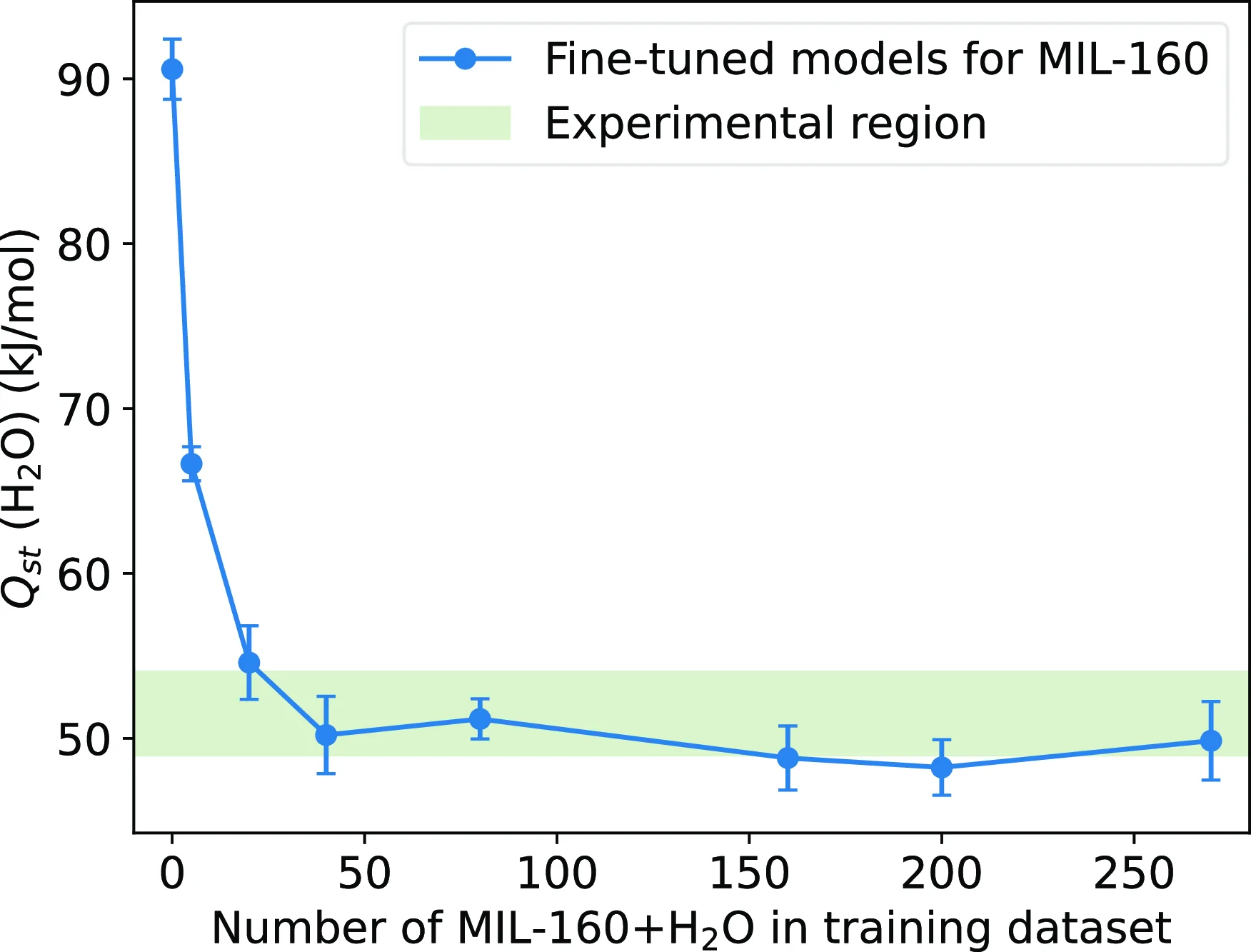
Predicted isosteric heat of adsorption, *Q*_st_(H_2_O), for MIL-160 as a function of the number of DFT-labeled configurations of MIL-160 with H_2_O used to fine-tune the MACE model. Each point stands for a fine-tuned model. In their training set, we kept the same number of pristine MIL-160 and isolated H_2_O configurations, but added a different number of configurations of MIL-160 with H_2_O. Error bars are the deviation of simulated *Q*_st_(H_2_O) from multiple MD simulations using the same fine-tuned model with the time scale of 20 ps. The green band is the range in which the experimental values of heat of adsorption are reported.^25–27^

**Figure 4:**
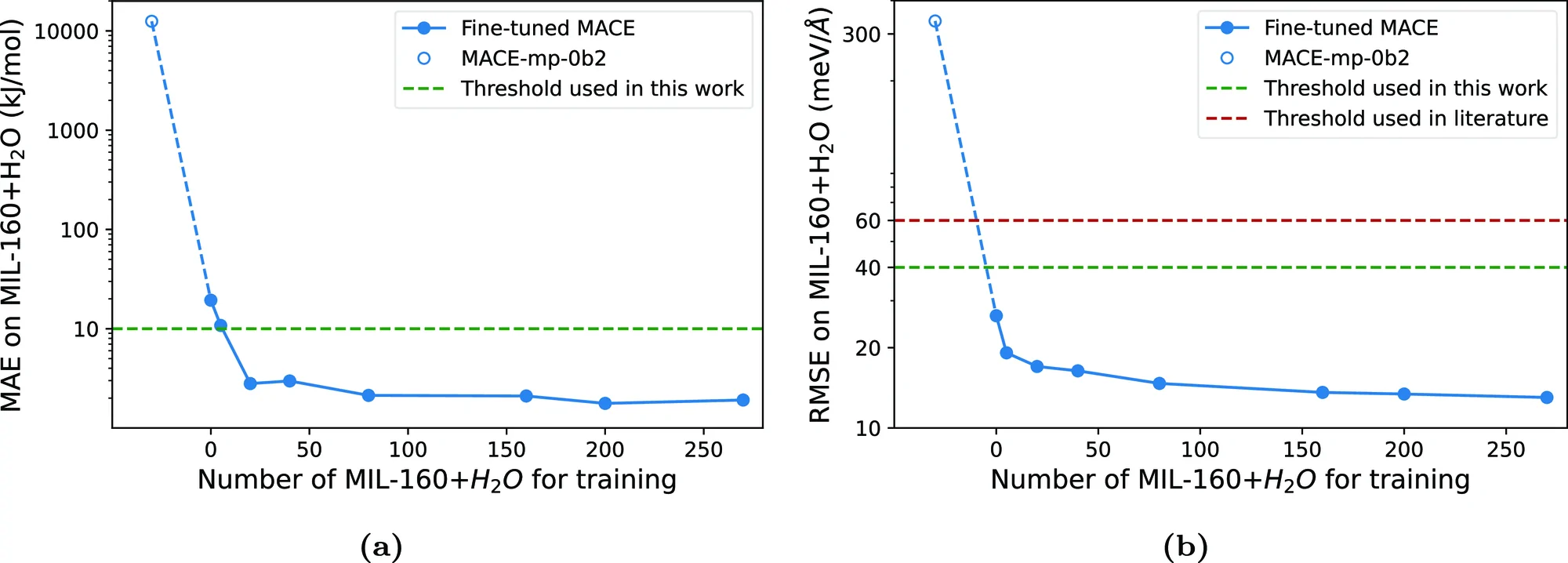
(a) Mean average errors (MAEs) on potential energies (unit in kJ mol^–1^ per configuration) of the test data set of MIL-160 with H_2_O as a function of the number of MIL-160 with H_2_O configurations in the training data set. (b) Root mean square errors (RMSEs) on atomic forces of the test data set of MIL-160 with H_2_O as a function of the number of MIL-160 with H_2_O configurations in the training data set. The green lines indicate the error thresholds below which the MLP yields reliable predictions of *Q*_st_. The red one is the threshold used in previous work.^30^

#### 2.2.2 Accuracy of the Total Energy

It is interesting to translate the results for the heat of adsorption of Figure 3 into the accuracy of our MLP’s representation of the DFT results. For this, we compute the mean absolute error (MAE) in energy and forces for our MLP as a function of the number of DFT labels added to our training set. We obtain this error by computing the difference between the energies and forces obtained from DFT and MACE. These errors are shown in Figure 4a for the energy and Figure 4b for the forces. The green line in these figures represents the criteria for the accuracy in the energy and forces we want to achieve to obtain a sufficiently accurate result for the heat of adsorption. We carried out similar tests on MFM-300 and KMF-1, and we obtained a similar threshold for the accuracy (see the Supporting Information for details).

Unless otherwise stated, energy errors are reported either as the total error of a complete periodic configuration, in kJ mol^–1^, or as the error normalized by the number of atoms, in meV atom^–1^. Here, one configuration corresponds to one periodic simulation cell; thus, an error in kJ mol^–1^ is expressed per mole of such simulation cells and is not normalized by the number of atoms. Force errors are reported in meV Å^–1^. The configuration-level energy error is used to assess its possible effect on the calculated *Q*_st_, whereas the per-atom error facilitates comparison with other machine-learning-potential studies.

If we use the MACE model out of the box, we see that the error for the energy is 3 orders of magnitude too large, while the error in the force is only a factor of 5 too large. One can rationalize this large difference between the error in the forces and energies by the way the MACE potential is trained. In this training, the loss function gives energy typically far less weight than forces. The energy error can also arise from inconsistencies in the absolute reference energies of the underlying DFT calculations. In particular, the training data set of the MACE-MP-0b model is generated using VASP, while our test data are computed with CP2K. These two codes use different basis sets and electronic structure representations, which do not have a large impact on the forces but can have a slight difference in the reference state for the energy of an atom. In addition, most of the training data comes from geometry optimization.^29^ These simulations typically give us a large amount of data on forces and, hence, data on changes in potential energy, but only a single number for the total energy. For the training of the forces, the absolute value of the energy is irrelevant. For most, if not all, properties of a single system, we only need differences in energy. However, for the heat of adsorption, we have to compute the difference in absolute energies between *different* systems, and in such a calculation, small errors in the atoms’ energies add up to a large number (see Supporting Information for details).

Figure 4a,b show that the error in the energies predicted by MACE is much larger than the error in the forces. Hence, if we compute the total energy of a MOF using MACE, we have a relatively large error in the total energy, but changes in the energy are predicted much more accurately. This has important consequences for the heat of adsorption, as this is not a systematic error. Because the presence of the water molecule changes the system and the large uncertainty in MACE’s estimate of the total energies, we obtain an error between the total energies of MACE and DFT that differs for MIL-160 with and without water. Hence, these errors do not cancel, and, therefore, this difference is a large factor in why MACE’s out-of-the-box prediction is performing so poorly for MIL-160.

These conclusions also hold for the other MOFs we studied in this work. We estimated the error by sampling 60 configurations for each pristine MOF and 60 for each MOF with a water molecule, and by comparing these MACE predictions with the corresponding DFT results, we obtained our estimate of the error in the total energies of both systems (see Supporting Information for details). Figure 5 shows the distribution of the error in *Q*_st_(H_2_O) caused by these errors in the total energy for the MACE-MP-0b2 foundation model on 420 Al-MOFs. We observe errors of up to 125 kJ mol^–1^ with an average error of 38 kJ mol^–1^ (see Supporting Information for details).

**Figure 5:**
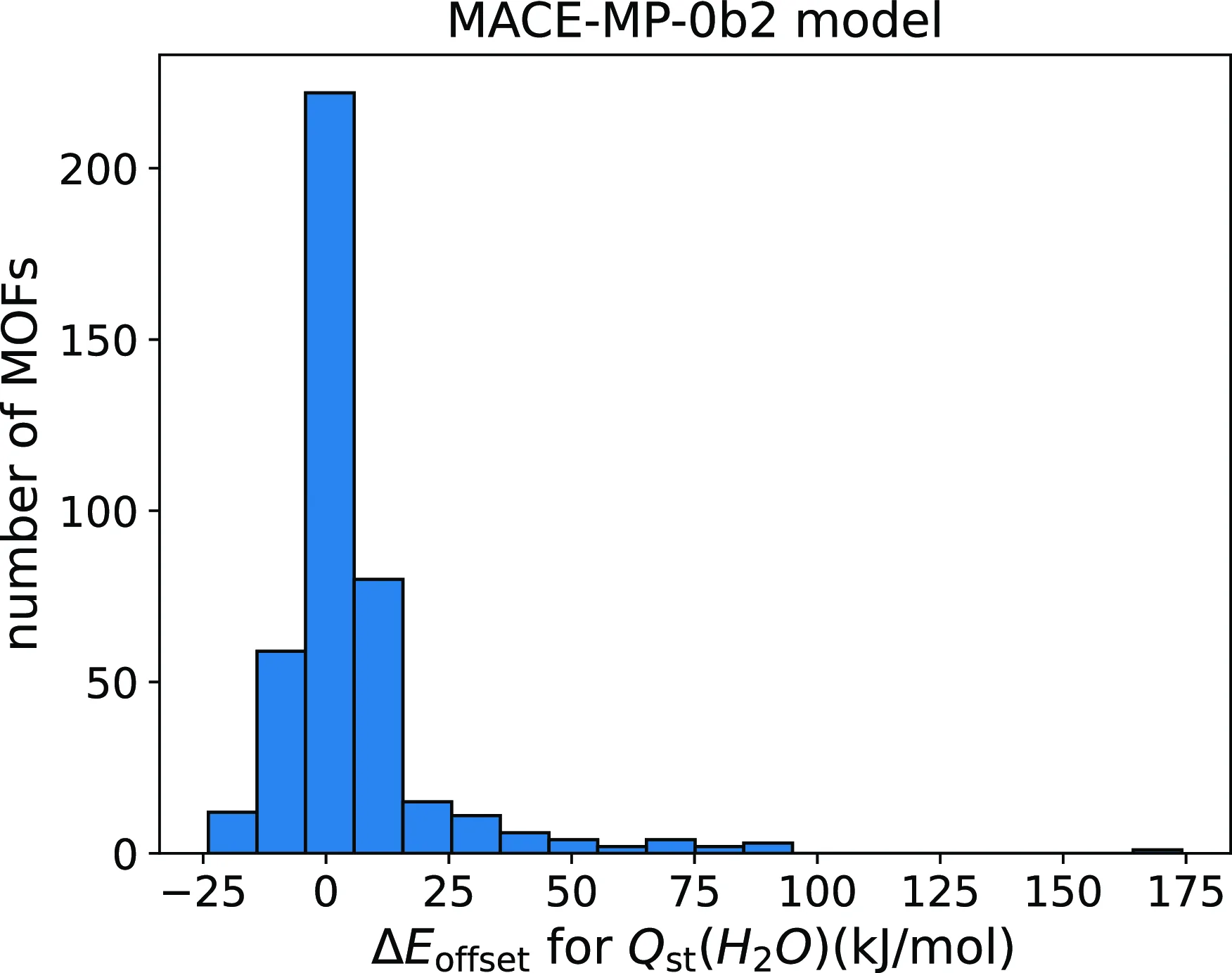
Distribution of errors in *Q*_st_(H_2_O) for the MACE-MP-0b2 model, which are caused by the different errors in the total energies of pristine MOFs and the MOFs with a H_2_O. We evaluate the MACE-MP-0b2 model on 420 Al-based MOFs, including the pristine MOFs and the MOFs with a H_2_O.

The way we estimate this error also provides a simple recipe for improving MACE’s prediction of the heat of adsorption. We estimated the error by sampling 30 configurations for each pristine MOF and 30 for the MOF with a water molecule beyond the test data set. The average difference between the total energies obtained by MACE and DFT provides an estimate of the error in the total energies of both systems. And, we can simply correct for these errors by adding a constant to MACE’s predictions of the total energies, i.e., applying a simple shift to the heat of adsorption. A similar approach was used by Wang et al.^30^ to compute acidity constants of organic molecules. Using this shift, the average errors in *Q*_st_(H_2_O) across 420 MOFs of the MACE-MP-0b2 model decrease from 38 kJ mol^–1^ to 9.5 kJ mol^–1^, which is a significant improvement. In addition to fine-tuning the MACE model on DFT data, this simple correction scheme provides an effective way to further improve the accuracy of heat-of-adsorption predictions.

#### 2.2.3 Fine-Tuning the MACE Potential

Our fine-tuning strategy aims to minimize the number of DFT calculations. For this, we use active learning, i.e., we run a molecular dynamics simulation with the current fine-tuned model (or the original MACE-MP-0b2 model for the first iteration) to explore the configuration space. From this simulation, we select the most diverse configurations and subsequently label them with DFT calculations^31^ (details of our training strategy are provided in Section 5.1.2).

In our study of MIL-160, we observed that small changes in hydrogen orientation within the MOF can have a large effect on hydrogen bonding with adsorbed water molecules. Hence, in our calculations, we need to fine-tune the MACE model for both the pristine MOF and this MOF with an adsorbed water molecule. As a first step, we train the MACE model on data from the pristine framework and isolated H_2_O. This data set is subsequently reused when further fine-tuning the model for MIL-160 with H_2_O. Figure 4a,b show that, for MIL-160 with H_2_O, adding this training set reduces errors in both energy and forces. For the forces, we have already achieved our desired accuracy, but for the energy, we need some additional DFT labels. In the Supporting Information, we show that the error can be further reduced by adding training data for liquid water.

The results in Figures 3 and 4a, suggest a threshold of MAE on energies (better than 10.0 kJ mol^–1^ for each configuration, i.e., better than 0.4 meV atom^–1^) for MIL-160 + H_2_O systems. Once the MLP error falls below this threshold, the fine-tuned MLP can yield a reliable *Q*_st_(H_2_O). For the forces, most studies used a root-mean-square error (RMSE) in atomic forces lower than 60 meV Å^–1^. This is sufficient to reproduce the ab initio molecular dynamics trajectories.^30^ For studies specific to MOFs, Lim et al.^21^ reported average errors of 20 kJ mol^–1^ in total energies and 85 meV Å^–1^ in atomic forces for 60 rigid MOFs with H_2_O or CO_2_. Elena et al.^32^ reported average errors of 13.2 kJ mol^–1^ in total energies and 14 meV Å^–1^ in atomic forces for 127 pristine MOFs. Our results for MIL-160 indicate that for the heat of adsorption, we need a more stringent criterion. We therefore adopt the following thresholds for our fine-tuning of the other Al-MOFs: for the energy an MAE better than 10.0 kJ mol^–1^ per configuration and for the forces an RMSE better than 40 meV Å^–1^.

## 3 Machine-Learning Potential for 420 Al-MOFs

In this section, we extend the fine-tuning of the MACE potential to a set of 420 Al-MOFs.

### 3.1 Data Set Construction

We constructed a data set comprising 420 Al-MOFs that contain only the most common organic elements (i.e., C, H, O, and N). This data set covers all Al-MOFs (only containing C, H, O, and N) in the well-known data sets, including the Quantum-MOF (QMOF) database,^33^ the CORE-MOF 2019 database,^34^ and the experimental data set provided by the Prisma project.^15^ To compare our predictions with experimental data, we also include those Al-MOFs for which adsorption data are reported in the context of water harvesting (e.g., CAU-10-X^22^ (X = H, NH_2_ and NO_2_), MFM-300,^23^ MIL-160,^25–27^ MIP-211 ^35^).

To clean our data set, we carried out the following steps. First, all structures are validated using the MOFchecker package^36^ to ensure that the MOF structure is chemically sound (e.g., no missing hydrogen atoms, no net charge, etc.). To eliminate duplicate structures, we initially use the mofdscribe package^37^ to convert MOF crystal structures into graph representations and identify duplicates. However, this approach cannot distinguish between structures that are identical but differ in unit-cell symmetry. To address this limitation, we utilize the Moffragmentor package^38^ to extract metal clusters and ligand information from Al-MOFs. The lists of metal clusters and ligands, as well as the isolated ligand CIF files, are available on Zenodo.^39^ Structures containing the same metal clusters and ligands are typically considered duplicates unless they have a different network. We manually checked these structures and removed 29 duplicated structures from our data set. The metal clusters and ligands in our data set are shown in Figure 6. All CIF files of the cleaned MOF structures are available on Zenodo.^39^

**Figure 6:**
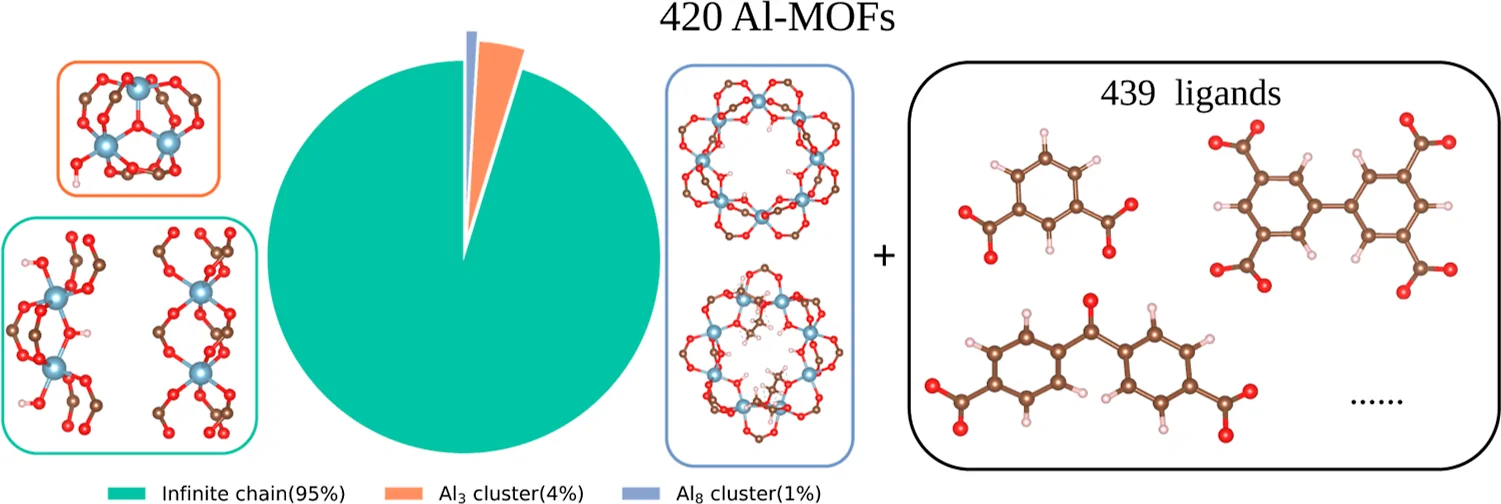
Compositions of the Al-MOFs data set. The data set comprises 420 Al-MOF structures built from three distinct types of metal node motifs (shown on the left) and 439 unique organic linkers (three examples shown on the right). In the structural diagrams, blue spheres represent aluminum atoms, red spheres represent oxygen atoms, brown spheres represent carbon atoms, and white spheres represent hydrogen atoms. Because some MOFs in the QMOF data set contain multiple ligands, the total number of unique ligands exceeds the number of Al-MOFs.

### 3.2 Model Training and Performance Evaluation

We first select 68 representative Al-MOFs from our data set to train a transferable MLP for the entire Al-based MOF data set. This subset includes Al-MOFs for which accurate experimental data is available (e.g., MIL-160, MFM-300, and CAU-10-X (X = NO_2_, NH_2_)), as well as 64 representative MOFs selected by the farthest point sampling method in the SOAP descriptor space from the whole data set. For these well-known MOFs, experimental heats of adsorption *Q*_st_(H_2_O) and Henry coefficients *K*_H_(H_2_O) have been reported, which allows us to directly validate the predictions of the MLP. We further assess the model’s transferability on an additional set of 352 Al-MOFs.

#### 3.2.1 Training on 68 Al-MOFs

For our training, we aim for an accuracy of the energy better than 10.0 kJ mol^–1^ per configuration and an force accuracy better than 40 meV Å^–1^. For this training, we used a similar workflow to that used for MIL-160, except that we now added the DFT labels for all 68 Al-MOFs to the training set (see Section 5.1.1 for details). Hence, the aim is to develop a single MLP model that works for all 68 Al-MOFs. We refer to this model as MACE-AlMOFs-68.

Figure 7 shows that for all 68 selected Al-MOFs the MACE-AlMOFs-68 model achieves an MAE for the energies of 0.108 meV atom^–1^ (1.9 kJ mol^–1^ per configuration) and RMSE on forces of 15.5 meV Å^–1^. Our estimate further improves slightly if we add the correction for the total energy.

**Figure 7:**
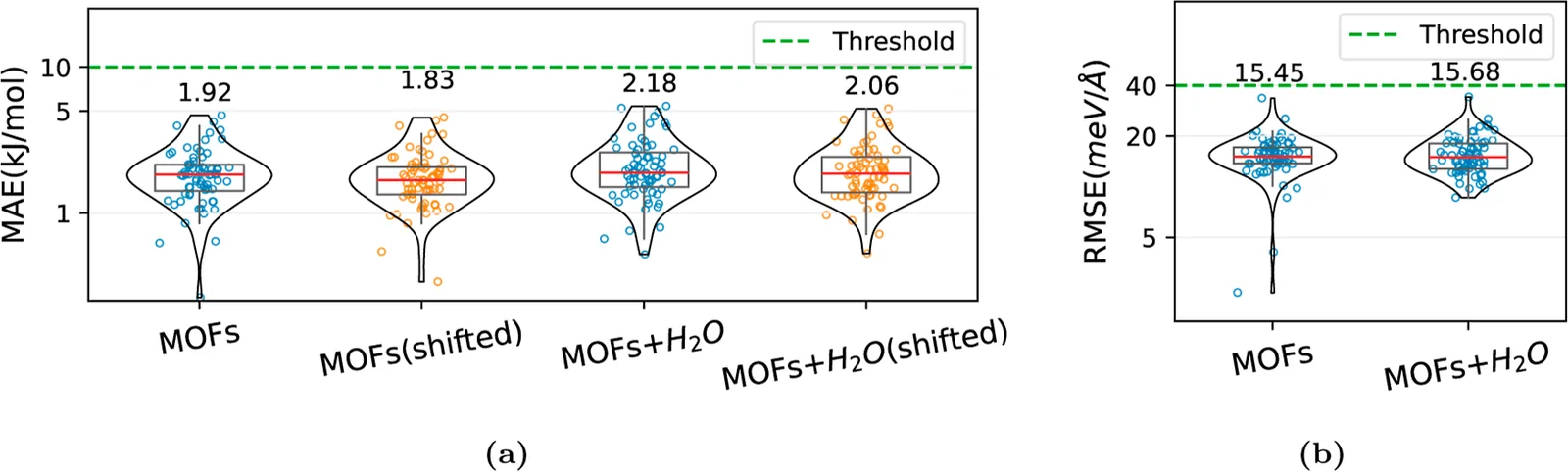
Errors of the MACE-AlMOFs-68 model for the 68 Al-MOFs that were used in the training set. (a) Energy errors in kJ mol^–1^ per configuration. (b) Force errors in meV Å^–1^. The test data set includes pristine MOFs and MOFs with one H_2_O molecule. Each point represents one type of Al-MOF. The value at the top indicates the average values across all points. Blue points correspond to direct predictions from the MACE-AlMOFs-68 model. The errors are computed by sampling 60 configurations sampled from 300 K *NVT* molecular dynamics simulations of the pristine MOF and the MOF with a water molecule, and comparing the forces and energies for the MACE-AlMOFs-68 model with those obtained by DFT. Orange points represent the predictions after the correction (see Section 2.2). The correction term is constant for each MOF.

#### 3.2.2 Transferability of the Model

An important question is how transferable our fine-tuned model is to other Al-MOFs not included in the training set. The results for the 352 out-of-sample MOFs are present in Figure 8. The average RMSE of atomic forces is 46.7 meV Å^–1^, and the average MAE of potential energies is 79 kJ mol^–1^ per configuration. For the forces, most of the Al-MOFs meet our threshold, but not for the energies.

**Figure 8:**
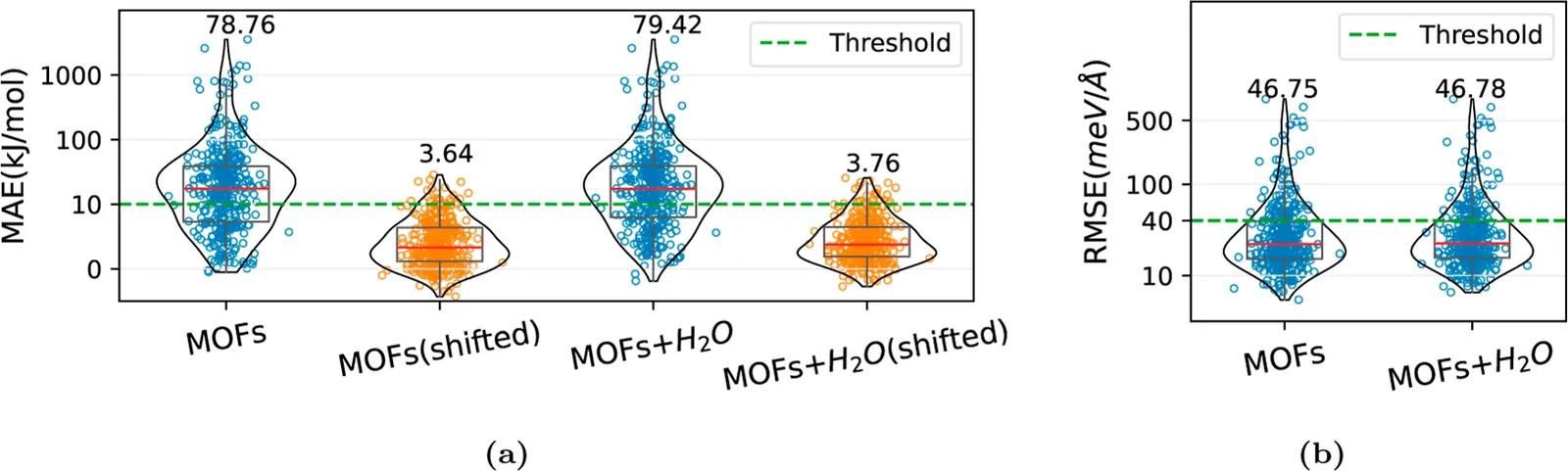
Errors of the MACE-AlMOFs-68 model on the test data of the 352 Al-MOFs that were not included in the training of the model. (a) Energy errors in kJ mol^–1^ per configuration. (b) Force errors in meV Å^–1^. The errors are computed by sampling 60 configurations sampled from 300 K *NVT* molecular dynamics simulations of the pristine MOF and the MOF with a water molecule, and comparing the forces and energies for the MACE-AlMOFs-68 model with those obtained by DFT. The test data set includes pristine MOFs and these MOFs with one H_2_O molecule. Each point represents one type of Al-MOF. The value at the top indicates the average values across all points. Blue points correspond to direct predictions from the MACE-AlMOFs-68 model. Orange points represent the predictions after the correction (see Section 2.2). The correction term is constant for each MOF.

The accuracy of our energies can be improved by correcting the predictions of the total energies. For this, we use DFT calculations to label 30 configurations for each of the 352 MOFs to estimate the energy offsets. To avoid possible correlations, we used a different set of configurations to compute the offset from the test set we used to compute the error. Figure 8 shows that with these energy corrections, the energy MAE is reduced to 3.7 kJ mol^–1^ per configuration (0.156 meV atom^–1^), and for most of the MOFs we now achieve an accuracy better than our threshold. In the Supporting Information, we show that with only 10 DFT calculations, a similar accuracy can be obtained.

#### 3.2.3 Additional Fine-Tuning

The transferability of the MACE-AlMOF-68 model indicates that it requires only minimal fine-tuning to generalize to the entire Al-MOFs data set. Therefore, we conduct one cycle of active learning for the remaining 352 MOFs and obtain a more robust model, referred to as MACE-AlMOFs-v1. We evaluate the MACE-AlMOFs-v1 model on the entire data set, comprising 420 MOFs. For 411 MOFs (both the pristine MOF and the MOF with H_2_O), we achieved the accuracy threshold. This model achieve the RMSE on atomic forces of 16.2 meV Å^–1^ and the MAE on potential energies of 2.23 kJ mol^–1^ per configuration (0.095 meV atom^–1^).

### 3.3 Transferability Indicators

Chemical diversity in the training data set is essential for constructing transferable MLPs with a minimal number of DFT-labeled configurations. Therefore, a quantitative and general metric is required to characterize the diversity of local chemical environments. In a previous study on CO_2_ adsorption in MIL-120, Jin et al.^31^ quantified chemical similarity using a simple distance metric in the SOAP descriptor space and employed this metric to guide diversity-based sampling of representative configurations. This strategy is motivated by a practical question: whether a new MOF that is not part of our sampling can be accurately described by our model. The underlying assumption is that if the chemical environments of the new MOF are sufficiently similar to those in our training set, we have some confidence that our MACE-AlMOFs-68 model will be sufficiently accurate without additional training.

We trained a simple three-layer neural network using SOAP descriptors to predict force errors of the MACE-AlMOFs-68 model on 352 out-of-sample Al-MOFs. Building upon SOAP distances between a new MOF and the training set, the model learns scalar weights that rescale these distances into a weighted distance matrix that directly predicts the force error of the MACE-AlMOFs-68 model on this MOF (see details in the Supporting Information).

Figure 9 shows that this model achieves 87% classification accuracy on the transferability of new MOFs within the test data set, which indicates that one can assess with reasonable confidence whether our MACE model can be used for an accurate MD simulation of an unseen MOF. When using our model for the heat of adsorption, one needs to perform about 10 DFT calculations to compute the shift in total energy and ensure energy accuracy. This model can also guide the selection of representative MOFs for sampling when extending the MACE model’s applicability to a new class of MOFs.

**Figure 9:**
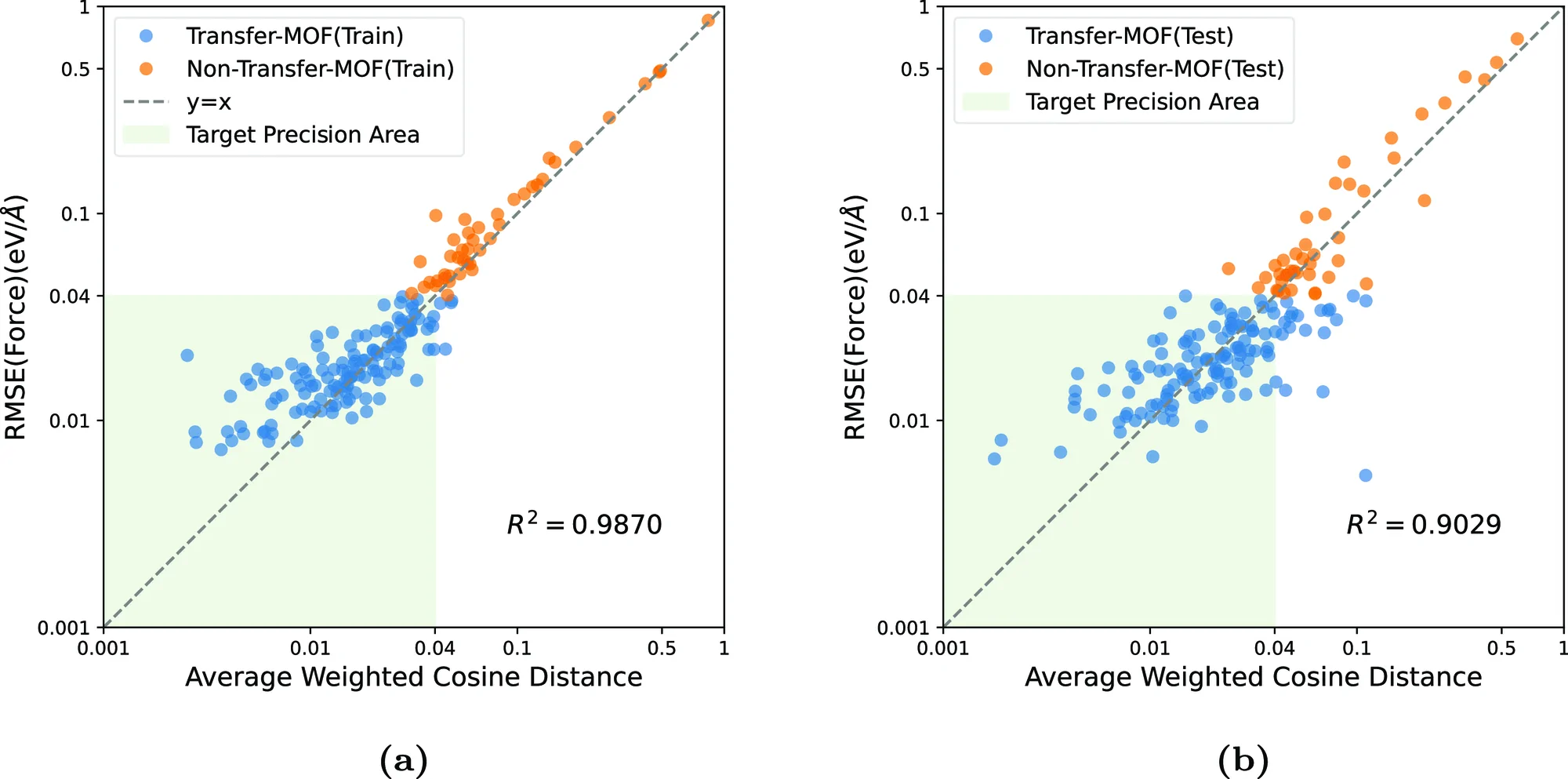
Comparison of force errors for MACE-AlMOFs-68 models across 352 MOFs, contrasting actual errors from the test data set with those predicted by our indicator model. (a) Results for the 176 MOFs included in the indicator model’s training data set. (b) Results for the 176 MOFs excluded from the training data set. Data points in blue and orange represent transferable and nontransferable MOFs, respectively, classified using a force RMSE threshold of 40 meV Å^–1^. Our indicator model achieves an 87% classification accuracy on the test data set.

### 3.4 Validation on Experimental Data

Accurate experimental data are limited, but we can make a comparison for the heats of adsorption and Henry coefficients of a series of well-known MOFs, including CAU-10-X (X = H, NO_2_, NH_2_), MFM-300, MIP-211, and MIL-160. In this series, CAU-10-H and MIP-211 are not part of the training set, but the MACE-AlMOFs-68 model can achieve our threshold on these two MOFs. The two examples also give us some (limited) insight into how well the model extrapolates.

As shown in Figure 10, the UFF force field significantly underestimates *Q*_st_(H_2_O) of MIP-211, MFM-300, KMF-1 and CAU-10-H compared to experimental values.^23,35,40^ We even see a change in the ranking, which makes predicted trends using the UFF of limited use. The MLP reproduces *Q*_st_(H_2_O) consistent with experimental results. Because the *Q*_st_(H_2_O) calculation is performed within a flexible framework, the uncertainty of simulated *Q*_st_(H_2_O) is higher compared with that in the rigid framework assumption.

**Figure 10:**
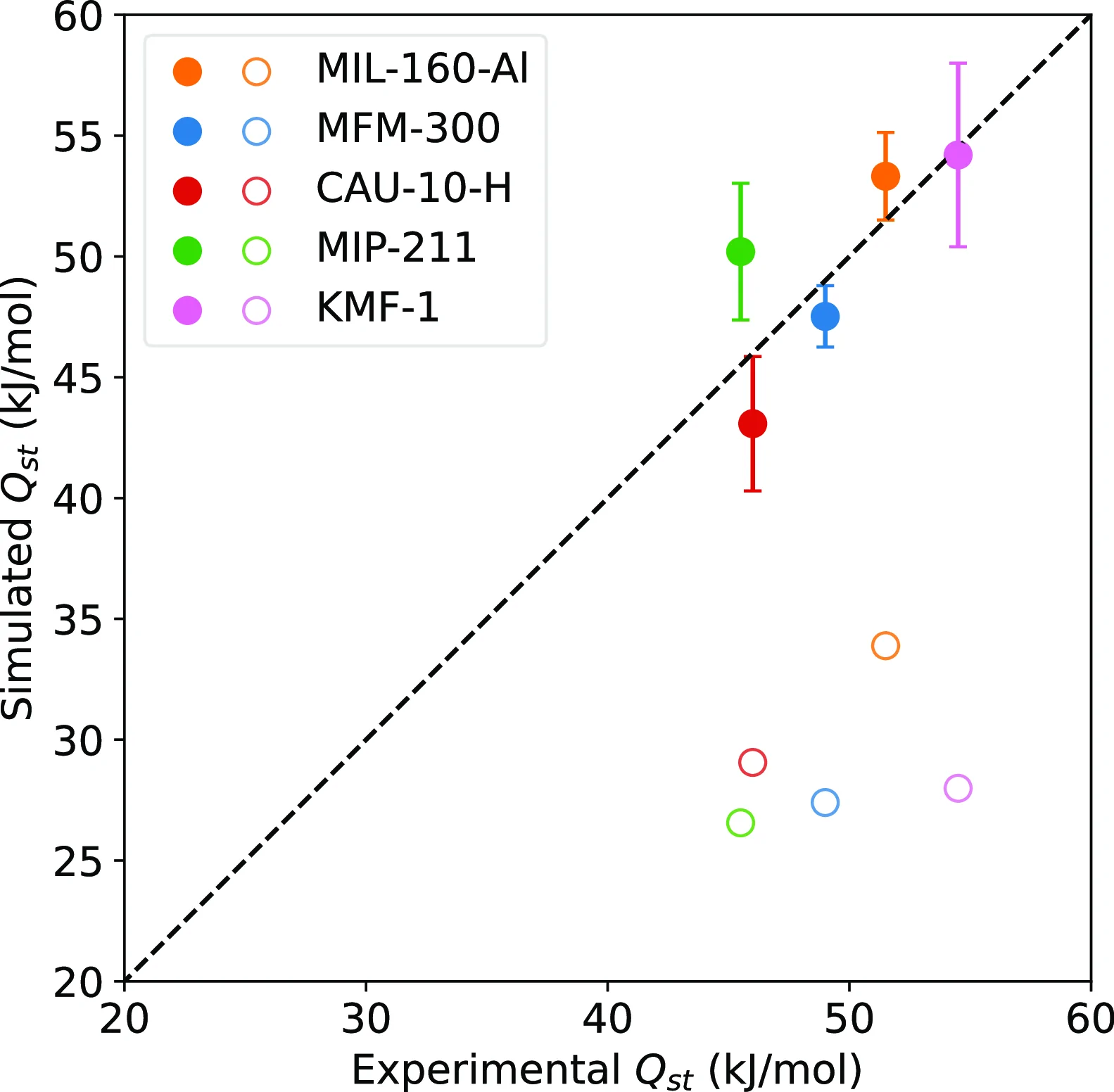
Comparison between the simulated zero-loading *Q*_st_(H_2_O) and the ones from experiments.^22,24,35^ The filled cycles are predicted by the fine-tuned MACE models, and the empty ones are predicted by the UFF. The *Q*_st_(H_2_O) of MIP-211 is derived from the water isotherms reported in the literature (see details in the Supporting Information).

Henry coefficients *K*_H_ are widely used as key performance indicators (KPIs) in screening studies of MOFs for gas adsorption, as they directly reflect adsorption isotherms at low pressure. We extracted *K*_H_(H_2_O) at 293.15 K of CAU-10-X (X = H, NO_2_, and NH_2_) from the experimental isotherms^22^ (see details in the Supporting Information), and simulated the Henry coefficients using both the UFF and the MACE-AlMOFs-68 model.

In Figure 11a, we compare the Henry coefficient as predicted by UFF with experimental data. In these calculations, we used assumptions similar to those in most screening studies:^4,6,21^ the framework is optimized using DFT, and in the simulation of the Henry coefficient, it is assumed to be rigid. As shown in Figure 11a, the UFF underestimates the Henry coefficients by one to 2 orders of magnitude. In addition, UFF predicts that the Henry coefficients of water in CAU-10-X do not depend on the functional group X (X = H, NO_2_, and NH_2_), whereas we experimentally observe an order-of-magnitude difference between CAU-10-H and CAU-10-NH_2_.

**Figure 11:**
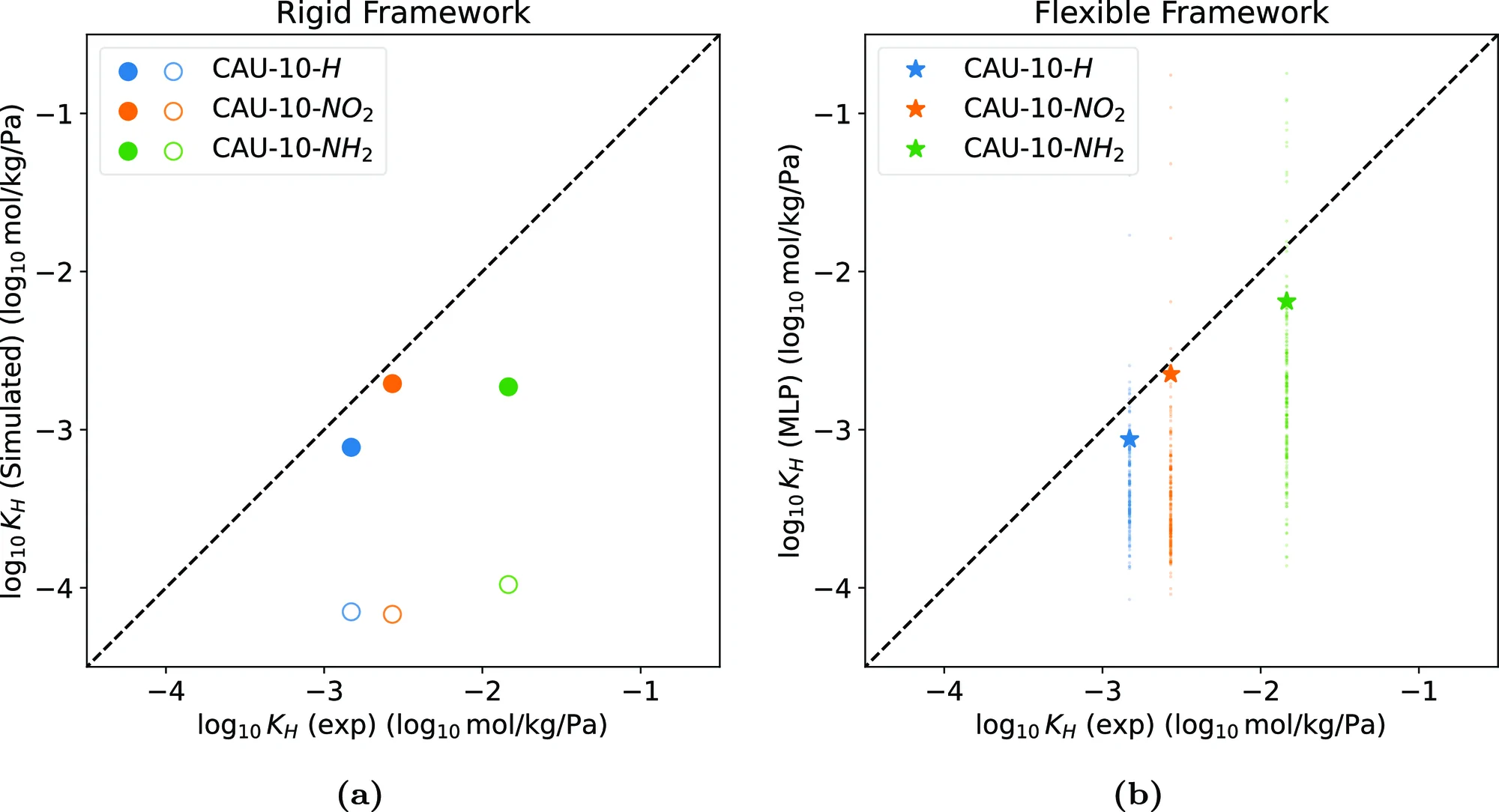
Comparison of simulated and experimental Henry coefficients of CAU-10-X (X = H, NO_2_, and NH_2_) at 293.15 K. Experimental values were extracted from the reported adsorption isotherms^22^ (see the Supporting Information for details). (a) Henry coefficients calculated using the MACE-AlMOFs-68 model (filled circles) and UFF (open circles) under the rigid-framework approximation. (b) Henry coefficients calculated using the MACE-AlMOFs-68 model with framework flexibility taken into account. Specifically, 100 configurations were randomly selected from *NVT* simulations of the pristine MOFs, and the corresponding *K*_H_(H_2_O) values were calculated under the rigid-framework approximation, as shown by the small filled circles. The averaged *K*_H_(H_2_O) values are reported as the final results and are shown as star symbols, thereby incorporating the effect of framework flexibility.

Figure 11a shows the predictions of the Henry coefficients if we use the same rigid framework assumption but replace UFF by our MACE-AlMOFs-68 model. We see a significant improvement in the numerical values. However, the model incorrectly predicts a lower Henry coefficient for CAU-10-NH_2_ than for CAU-10-NO_2_, whereas experimental measurements indicate that the Henry coefficient of CAU-10-NH_2_ is approximately 1 order of magnitude higher. Figure 11b shows the results when we use a fully flexible MOF. We now obtain results that agree very well with the experimental data. For CAU-10-H and CAU-10-NO_2_, the DFT-optimized configurations have binding sites for H_2_O that are very close to the optimal ones. Hence, we observe only a small change when using a fully flexible model. In CAU-10-NH_2_, a H_2_O molecule can interact with both the NH_2_ group and the μ_2_-OH group within the AlO_6_ cluster, but only if the two groups point in the right direction, which is not the case in the DFT-optimized structure.

The *K*_H_(H_2_O) calculation is quite sensitive to the configurations, especially in the systems with multiple strong hydrogen bonds like CAU-10-NH_2_. For CAU-10-H and CAU-10-NO_2_, the DFT-optimized configurations are fortunately close to the correct values. In CAU-10-NH_2_, the H_2_O can interact with both the NH_2_ group and the μ_2_-OH group within AlO_6_ cluster, which replies on the relative positions between NH_2_ and μ_2_-OH groups.

In summary, our model predicts *Q*_st_(H_2_O) and *K*_H_(H_2_O) values that are consistent with the available experimental data. Notably, CAU-10-H and MIP-211 were not included in the training data set and therefore serve as out-of-sample examples. For both MOFs, once the model satisfies our accuracy thresholds on DFT-labeled configurations, it also provides reliable predictions of *Q*_st_(H_2_O) and *K*_H_(H_2_O).

### 3.5 Comparison with UFF

The *Q*_st_(H_2_O) and Henry coefficient *K*_H_(H_2_O) are widely used as key performance indicators (KPIs) in previous screening studies^4,6^ for water-based applications, typically computed using the UFF force field under the rigid-framework approximation. In this work, we compare the predictions of these two properties with our MACE-AlMOFs-v1 model.

As show in Figure 12, the UFF force field systematically underestimate the *Q*_st_(H_2_O) and *K*_H_(H_2_O) for most MOFs. In comparison with the MACE-AlMOFs-v1 predictions, the UFF underestimates *Q*_st_(H_2_O) by more than 3.5 kJ mol^–1^ for 91% Al-MOFs and more than 10 kJ mol^–1^ for 74% Al-MOFs. A similar trend is observed for *K*_H_(H_2_O), where UFF predicts values that are more than 1 order of magnitude lower for 72% of the structures. In addition, the UFF results are confined to a significantly narrower range than MLP predictions. As a result, UFF fails to distinguish between materials with distinct adsorption characteristics, assigning similar *K*_H_(H_2_O) values to chemically diverse frameworks, as exemplified by the CAU-10-X series (see details in Figure 12).

**Figure 12:**
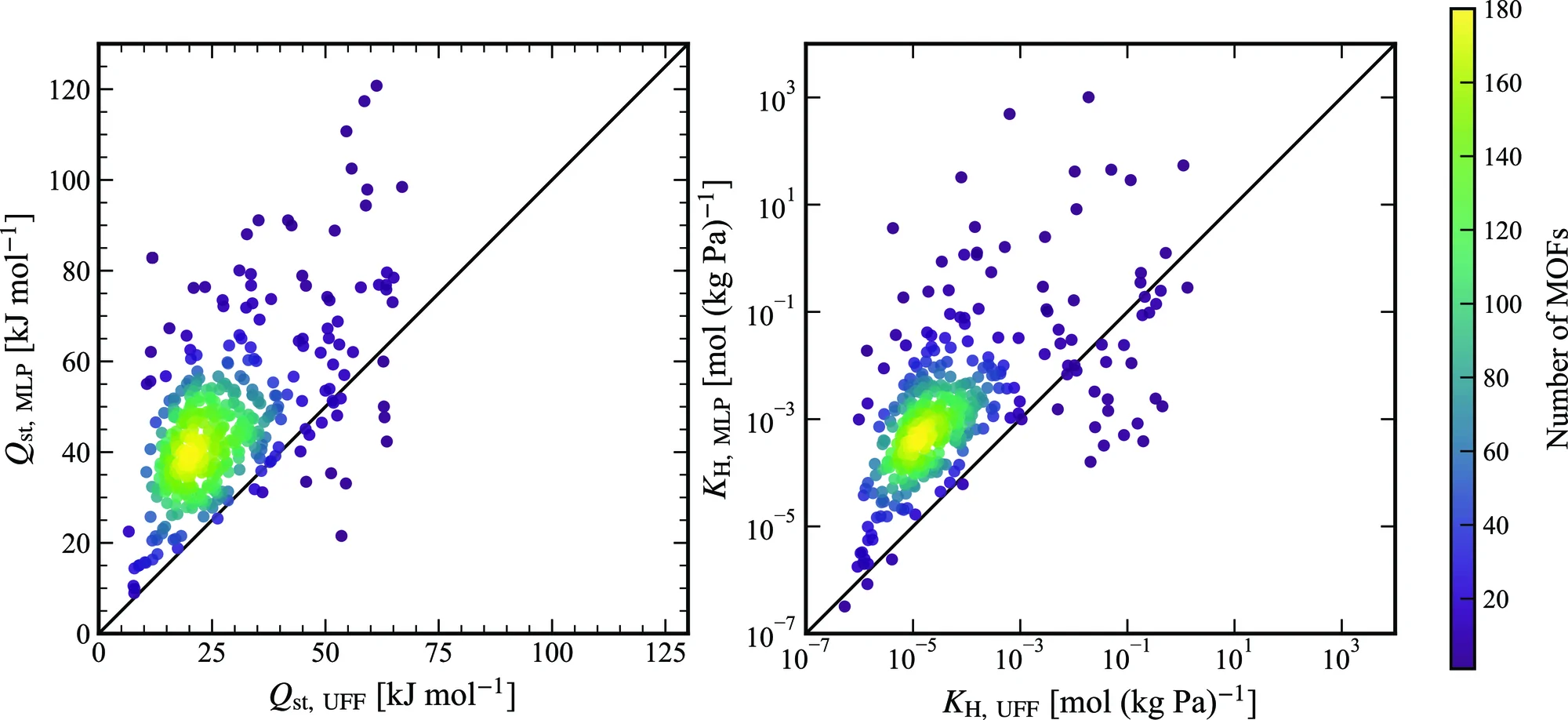
Comparison of *Q*_st_(H_2_O) (left) and *K*_H_(H_2_O) at 293.15 K (right) across 410 MOFs, as predicted by the MACE-AlMOFs-v1 model and the UFF force field.

To further analyze the impact of different models on screening outcomes, we examine the top 10 MOFs with the highest water affinity, identified based on the *K*_H_(H_2_O) values predicted by the MACE-AlMOFs-v1 and UFF models. The UFF-based ranking predominantly selects MOFs containing AlO_3_ metal nodes (see the structure in Figure 6), which provide open metal sites that can directly coordinate water molecules. Specifically, nine of the top-ranked structures from the UFF contain such open metal sites. The MACE-AlMOFs-v1 model also identifies five MOFs with AlO_3_ clusters among its top ten candidates, indicating partial overlap with the UFF screening results. The corresponding simulation data are available on Zenodo.^39^

## 4 Structure Optimization

The optimal configurations of MOFs are crucial for accurately simulating gas adsorption within MOFs. The rigid-framework assumption is widely used in simulations and strongly depends on whether the structure is fully optimized, i.e., a global energy-minimum configuration. For example, if one wants to compute the binding energy of H_2_O or CO_2_ using DFT, one needs to subtract the minimum energy configuration of the empty MOF, and if one does not have a fully optimized structure, one can make significant errors.^41^ Also, the predictions of the heat of adsorption and Henry coefficients can be quite sensitive to the details of these minimum-energy configurations.^14,42^

The structures in our Al-MOFs data set are taken from previous studies,^15,33,37^ in which the researchers have already optimized them using DFT calculations. The DFT functionals used in the previous studies are the same as those used for our training data set. Hence, one would expect that if we were to optimize our structures using our MACE-AlMOFs-v1 model, we would obtain the same minimum-energy configurations.

However, while running an *NVT* MD simulation with our MACE-AlMOFs-v1 on a MOF from the QMOF database (qmof-340b60d), using the minimum-energy conformation from the QMOF database as the initial configuration, we noticed a transition (see Figure 13b). The energy profile exhibits two distinct plateaus, where the MACE-AlMOFs-v1 model predicts that the second plateau has a significantly lower energy (Δ*E* = 350 kJ mol^–1^). We extracted the five lowest-energy configurations from this MD trajectory and reoptimized them using the MACE-AlMOF-v1 model (see Figure 13c). We then computed the energy using DFT, and the lowest-energy structure was 331.8 kJ mol^–1^ lower in energy than the one from the QMOF database.

**Figure 13:**
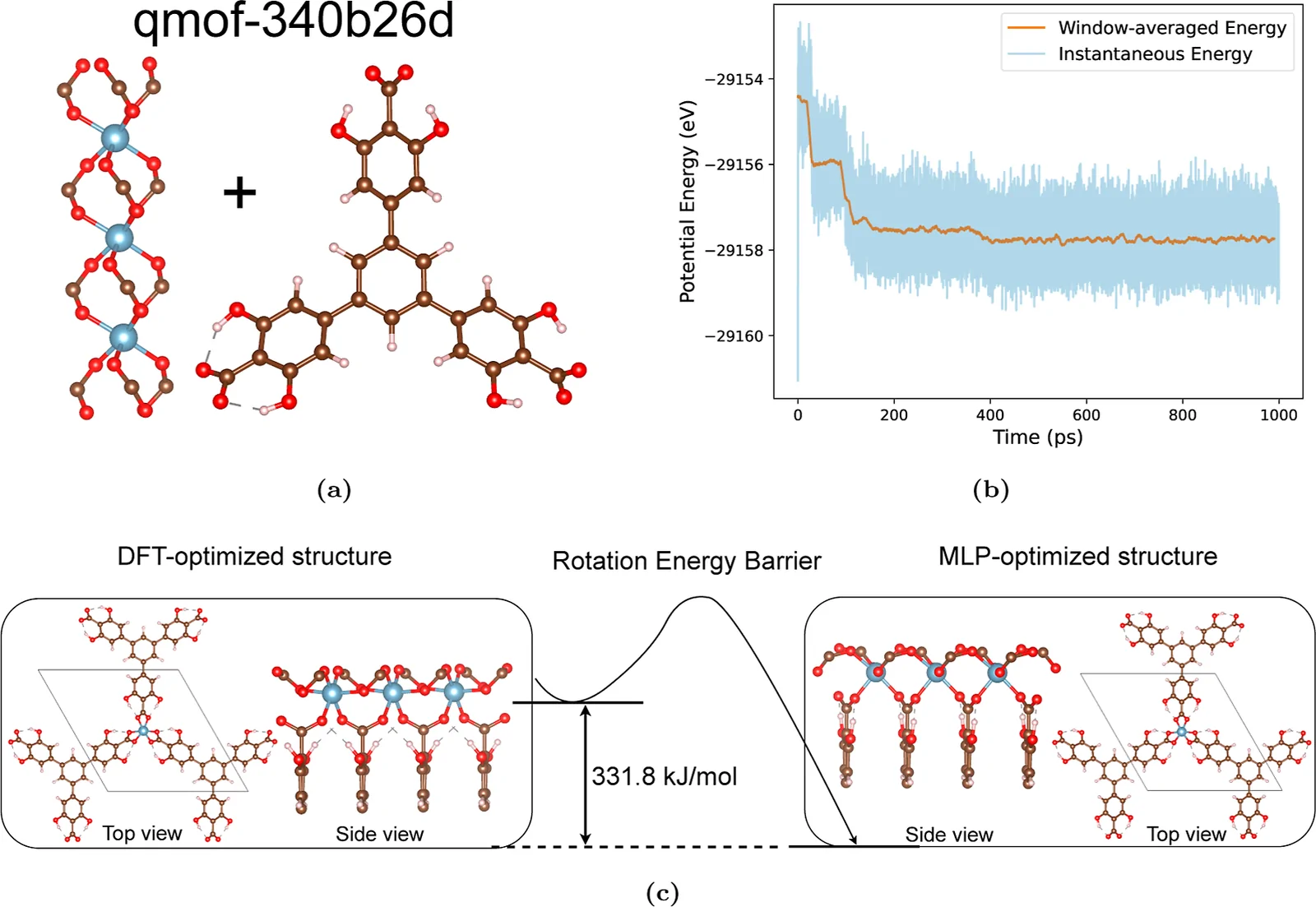
(a) The building blocks of qmof-340b60d. (b) Potential energy as a function of simulation time in the MD simulation of qmof-340b60d (without water) at 300 K in the *NVT* ensemble (blue). The orange curve shows the energy profile smoothed with a 1 ps sliding-window average. (c) The structural transition from the initial DFT-optimized structure (left) to the one optimized by the MACE-AlMOF-v1 model (right). The energy decrease of 331.8 kJ mol^–1^ is calculated using DFT. In the side view of the MOF, the three benzene rings of the ligand are omitted, and only one benzene ring is shown to highlight the hydrogen bond network. The blue spheres represent aluminum atoms, red spheres represent oxygen, brown spheres represent carbon, and white spheres represent hydrogen.

The qmof-340b60d is composed of aluminum rod-like secondary building units connected with the H_3_BTB linkers decorated with six hydroxyl groups (see Figure 13c). The infinite AlO_6_ chains are only bridged by the carboxyl groups of the linkers, without μ_2_-OH groups. This MOF structure features large hexagonal channels in which the hydroxyl groups can form strong hydrogen bonds with the AlO_6_ clusters. The structure reported in the QMOF databases has a different hydrogen-bond network compared to our minimum-energy configuration. This rearrangement results in an energy decrease of 331.8 kJ mol^–1^, involving a 60° collective rotation of the benzene ring within the ligands. As shown in Figure 13b, this rotation takes 100 ps in the MD simulation, which is prohibitively expensive for the large-scale DFT optimization of MOF data sets.

This observation motivated us to reoptimize 415 Al-MOFs in our data set using a similar procedure with the MACE-AlMOF-v1 model. For each MOF, we performed 300 ps of MD at 300 K, followed by geometry optimization of the five lowest-energy configurations. The DFT-calculated energies of the resulting optimal configurations were then compared with those from the original (DFT-optimized) data sets.

For 7 MOFs, we found that the optimal structures from the MACE-AlMOF-v1 model have a higher DFT energy than the original structure. However, the energy difference is relatively small, 0.5 kJ mol^–1^ to 5.3 kJ mol^–1^, and lies within the expected error. For the remaining 408 MOFs, Figure 14 shows that the MACE-AlMOF-v1 model yielded significantly lower energies. The optimal energy decreases by more than 20 kJ mol^–1^ for 68% of Al-MOFs and by more than 100 kJ mol^–1^ for 34% of Al-MOFs. Therefore, the MACE-AlMOF-v1 model can search for global energy minima for all 415 Al-MOFs, which were previously optimized using DFT in prior work.^15,33,37^ One should, however, realize that our method, like DFT optimization, does not guarantee that we have the global minimum energy for all MOFs. Therefore, we can only conclude that our estimates are better than the ones obtained previously. All optimized configurations are available on Zenodo.^39^

**Figure 14:**
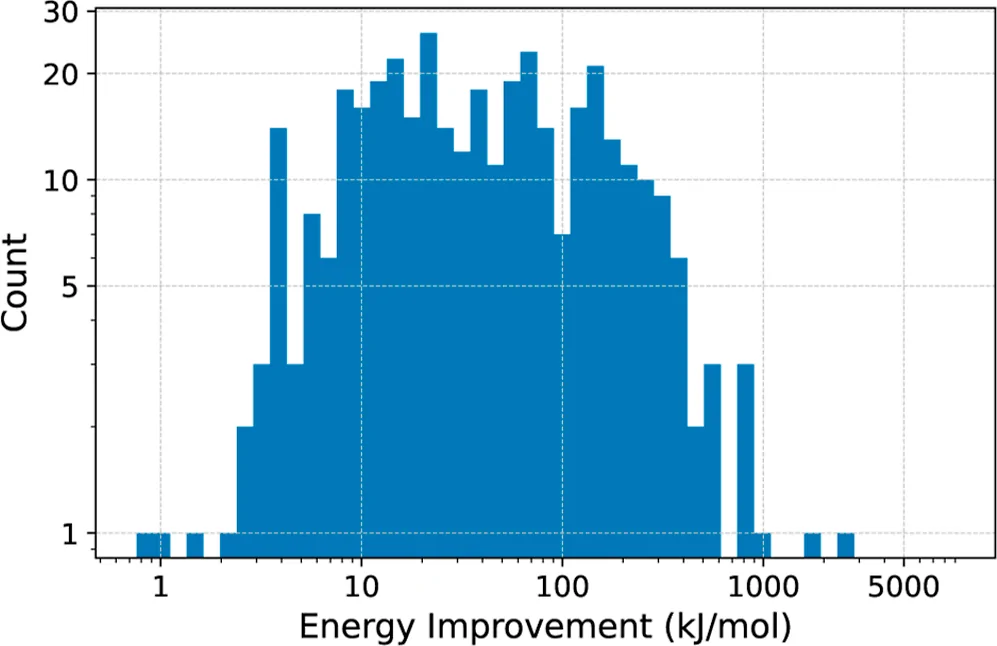
Histogram of energy improvement for the configurations optimized with the MACE-AlMOF-v1 model, compared with those optimized by DFT calculations. Both energy improvements and the number of MOFs are shown on a logarithmic scale.

A geometry optimization using DFT typically requires 10–200 DFT calculations. Sriram et al.^43^ used these DFT calculations to train a machine learning potential (MLP). Our results suggest an alternative strategy: to perform the geometry optimization using the machine-learning potential. This has an important practical advantage: with far fewer DFT calculations, one can obtain equivalent results. In a DFT optimization process, one computes the energies of many configurations that are too similar to provide much information to the training, whereas in our approach, we select a set of the most diverse configurations from long MD simulations using the MLP. As shown in Figure 13c, these long MD simulations can explore a much broader configuration space than direct DFT-based sampling, and the RMSEs on atomic forces (lower than 40 meV Å^–1^ ^30^) can guarantee ab initio accuracy of the simulations.

## 5 Method

### 5.1 MLP Training

#### 5.1.1 Fine-Tuning Protocol

Fine-tuning was performed on the pretrained MACE-MP-0b2 (medium) model^17^ using the *mace_torch* package. Therefore, the architecture was inherited from the pretrained model: the “medium” size indicates that this model uses two message-passing layers. The local chemical environments are defined by the cutoff radius of 5 Å.

A two-stage fine-tuning strategy was adopted to balance the learning of contributions from energy, force, and stress. In the first stage, the loss weights for energy, force, and stress were set to 1:10:1, placing greater emphasis on force learning to mitigate overfitting. In the second stage, the weights were adjusted to 1000:1:10 to focus on accurate energy prediction. Optimization was performed with a batch size of 8, an initial learning rate of 1 × 10^–3^, a reduced learning rate of 1 × 10^–4^ in the second stage, and a weight decay of 5 × 10^–7^.

#### 5.1.2 Active Learning Workflow

In the initialization stage, the pretrained MACE-MP-0b model, which exhibited excellent molecular dynamics (MD) stability,^44^ was employed for MD sampling at 300 and 500 K in the canonical ensemble (constant number of particles, volume, and temperature; *NVT*).

Next, the MACE model was fine-tuned using the same hyperparameters but with different random seeds, yielding four models. We selected 10 representative configurations from the training data set using *K*-means clustering in the SOAP descriptor space. For each configuration, we performed an independent 30 ps MD simulation. The four models were then used to evaluate model deviations for each configuration in MD trajectories. For a configuration *R*_*t*_, the model deviation ϵ_*t*_ was quantified as the maximum atomic force deviation^45^

2 $$\epsilon_{t}=\max_{i}\sqrt{{\left\langle {∥F_{k,i}\left(R_{t}\right)-{\left\langle F_{k,i}\left(R_{t}\right)\right\rangle }_{k}∥}^{2}\right\rangle }_{k}}$$

where *k* indexes the models, *i* indexes the atoms, **F**_*k*,*i*_(*R*_*t*_) is the predicted force vector, ⟨·⟩_*k*_ denotes the average over models, and ∥·∥ is the Euclidean norm.

Configurations with ϵ_*t*_ exceeding 60 meV Å^–1^ were considered to be in regions of configuration space that the model does not encounter. From this set of high-deviation configurations, we again applied *K*-means clustering to choose a subset of representative configurations, which were then labeled by DFT calculation and added to the training data set. Finally, the MACE-AlMOFs-68 model is fine-tuned on 43,375 configurations, and the MACE-AlMOFs-v1 model is fine-tuned on 166,925 configurations. The test data set is sampled from additional MD simulations at 300 K in the *NVT* ensemble.

### 5.2 DFT Calculation

The Quickstep code of the CP2K^46^ software was used to perform DFT calculations for labeling configurations. These calculations were performed using the Perdew–Burke–Ernzerhof (PBE) functional within the generalized gradient approximation (GGA),^47^ with Grimme’s D3 corrections for van der Waals interactions.^48^ To reduce the basis set superposition error arising from the use of Gaussian-type basis functions in CP2K, a relatively large TZV2P-MOLOPT basis set was employed. All DFT calculations employed the Γ-point approximation, which does not work for a too small unit cell. Therefore, unit cells of MOFs are enlarged to satisfy a cutoff radius of 10 Å.

A good initial guess of the wave function can significantly reduce the number of iterations for self-consistent field (SCF) convergence. For configurations in the same system, like MOF or MOF + H_2_O, we can use the wave function of the last configuration as the initial guess to accelerate the DFT calculations of the current configuration. The workflow was developed in the previous work.^49^ This strategy can reduce the total DFT single-point calculation time by 48% for 100 IRMOF-8 configurations compared with the ones without initial guesses. The CP2K input script can be found in Zenodo.^39^

### 5.3 Machine-Learning-Potential Simulations

#### 5.3.1 Structure Optimization

As discussed in Section 4, the optimal configurations are essential for the simulation of gas adsorption within MOFs, and our fine-tuned MACE model can find configurations with lower DFT-labeled energy compared with those optimized by the DFT calculation in previous works.^15,33,37^ We first use the MACE-AlMOF-v1 model to run 300 ps MD simulations at 300 K in the *NVT* ensemble, which can avoid local energy minima. From the simulation, we choose the five lowest-energy configurations and use the MACE-AlMOF-v1 model to optimize these configurations. The convergence criterion is the maximum atomic force below 20 meV Å^–1^, which is commonly used in previous studies.^46^ Considering the average RMSE on atomic forces of the MACE-AlMOF-v1 on 420 Al-MOFs is 23 meV Å^–1^, this convergence criterion is also suitable for the MLP. The Broyden–Fletcher–Goldfarb–Shanno (BFGS) algorithm is used for geometry optimization. All simulations are done in the *ase* package.^50^

#### 5.3.2 Henry Coefficient Calculation

The Henry coefficient *K*_H_ is calculated by the Widom insertion method^51^ within a rigid MOF. Our implementation was based on the *DAC-SIM* package^21^ but corrected two issues in the original code: incorrect framework energies after automatic cell scaling and nonuniform sampling of molecular orientations caused by the wrong generation of random rotation matrices in three-dimensional space.

The Henry coefficient considering framework flexibility is the average value of *K*_H_(H_2_O) for a series of pristine MOF configurations, which are randomly selected from MD trajectories. A similar approach was previously used by Witman et al.^14^ for noble gas adsorption. The systematic energy offsets, i.e., $\Delta {\mathrm{E̅}}_{MOF+H_{2}O}$ and $\Delta {\mathrm{E̅}}_{MOF}$ can be reduced in the Henry coefficient calculation. The specific equation is shown below, and *K*_H_^′^ is the Henry coefficient with the correction.

3 $$K_{H}^{\prime }=K_{H}\;\exp\left(\frac{\Delta {\mathrm{E̅}}_{MOF}+\Delta {\mathrm{E̅}}_{H_{2}O}-\Delta {\mathrm{E̅}}_{MOF+H_{2}O}}{RT}\right)$$

For *K*_H_(H_2_O) with the rigid-framework approximation, 5 × 10^4^ Widom insertion cycles are performed with the DFT-optimized configuration. For the Henry coefficient considering framework flexibility, 150 configurations are selected from the *NVT* MD trajectory at 293.15 K, and for each configuration, 2 × 10^4^ Widom insertion cycles are performed to ensure statistical convergence. If the minimum distance between the inserted water and the framework atoms is smaller than 1.5 Å, a large positive energy penalty is assigned directly instead of evaluating the energy using the MLP.

#### 5.3.3 Calculation of the Heats of Adsorption

The zero-loading *Q*_st_(H_2_O) (see eq 1) is widely used for screening studies,^4,6^ which means that the interaction between H_2_O molecules are almost zero. We calculate the interaction energy between the two water molecules separated by 10 Å, which is 0.32 kJ mol^–1^. Due to the requirement of Γ-point approximation, all unit cells are enlarged to satisfy the cutoff radius of 10 Å. Therefore, the *Q*_st_(H_2_O) is calculated for one H_2_O per unit cell, in which the H_2_O–H_2_O interaction can be ignored.

#### 5.3.4 Hybrid MD/MC

The ensemble-averaged energies in eq 1 for pristine MOFs were obtained from 1000 ps MD simulations at 300 K in the *NVT* ensemble. However, for MOFs containing H_2_O, conventional MD simulations may suffer from insufficient configurational sampling because of the diffusion of H_2_O within some specific MOFs.

For example, CAU-10-H with H_2_O has two types of water-binding sites. The water molecule is easily trapped at a single binding site for more than 50 ps without visiting other binding sites. Although substantially longer MD simulations (e.g., 3000 ps) can eventually improve sampling, such simulations remain computationally expensive even when using MLPs for hundreds of MOFs. Furthermore, in MIP-212,^15^ there are two types of pores connected by narrow channels that hinder the diffusion of H_2_O between them. In these systems, conventional MD simulations cannot efficiently sample the configurational space with the correct ensemble distribution.

To improve sampling efficiency, we introduced reinsertion Monte Carlo (MC) moves during the MD simulations. The ensemble-averaged energies of MOFs containing H_2_O were therefore obtained from hybrid MD/MC simulations with a total simulation time of 800 ps. During the simulation, reinsertion MC moves were attempted every 1000 MD steps, with 300 trial insertions performed at each MC stage.

During MD simulations, the framework may relax toward lower-energy minima, leading to substantial changes in the ensemble-averaged energy, exceeding 100 kJ mol^–1^ as shown in Figure 14. Such large variations would significantly affect the heat of adsorption. To avoid this issue, all structures are first optimized using the method in Section 5.3.1 before MD simulations. To further ensure convergence, each trajectory (for both pristine MOFs and MOFs with H_2_O) is divided into three blocks, and the ensemble-averaged energies are evaluated for each block. If the maximum energy difference between block averages exceeds 15 kJ mol^–1^, we identify the ten lowest-energy configurations from the trajectory, perform additional geometry optimizations, and restart the simulations from the most stable structure. This procedure is repeated until the energy profile converges. After at most three iterations, this workflow yields converged ensemble-averaged energies for all 410 pristine MOFs and MOFs containing H_2_O.

All *NVT* simulations of pristine MOFs and MOFs containing H_2_O were performed using the *ase* package.^50^ A time step of 1 fs was used throughout. The temperature was controlled using a Langevin thermostat with a damping time of 10 fs.

### 5.4 Classical Force-Field Simulations

In the simulations using the UFF force field, we assume that the MOF framework is rigid. The interactions of the atoms in the MOFs with the adsorbed water molecules are described by van der Waals (vdW) interactions and Coulomb interactions. The vdW interactions are described with the Lennard-Jones potential. The Lennard-Jones parameters of MOFs are inherited from the UFF force field, and the ones of water molecules come from the TIP4P/2005 force field.^52^ The partial charges on water molecules are inherited from the TIP4P/2005 force field, and those on the MOF atoms are derived by the DDEC method.^53^ The Ewald summation is used to calculate the electrostatic interactions. For the Lennard-Jones interactions, we use a cutoff distance of 12.8 Å as suggested by Jablonka et al.^54^ The entire potential is shifted based on its value at the cutoff distance to avoid the truncation error. Additionally, a tail correction is applied. The standard Lorentz–Berthelot combining rules are applied to obtain the Lennard-Jones parameters ϵ_*ij*_ and σ_*ij*_ between different types of atoms.

The *Q*_st_(H_2_O) come from the *NVT* MC simulations, and the Henry coefficient *K*_H_ is also calculated by the Widom insertion method^51^ within a rigid MOF. All simulations are done in the RASPA molecular simulation software.^55^

## 6 Concluding Remarks

Accurately describing water adsorption in metal–organic frameworks remains a major challenge in molecular simulation. Although classical force fields have proven successful for many gas adsorption applications, they often fail to capture the strong, highly directional hydrogen-bonding interactions that govern water adsorption in MOFs. In this work, we demonstrate that machine-learning potentials offer a practical way to overcome these limitations. By fine-tuning a pretrained MACE foundation model, we obtain DFT-level predictions of water adsorption thermodynamics while explicitly accounting for framework flexibility.

A central finding of this work is that framework flexibility is not a secondary effect but a prerequisite for accurately predicting water adsorption properties. Comparisons with experimental heats of adsorption and Henry coefficients show that even small reorientations of hydrogen-bonding groups within the framework can substantially alter adsorption energetics. These effects are absent in conventional rigid-framework simulations but are naturally captured by the machine-learning potential.

Using MIL-160 as a benchmark system, we established practical accuracy criteria for predicting adsorption thermodynamics, namely an energy MAE below 10 kJ mol^–1^ per configuration and a force RMSE below 40 meV Å^–1^. These thresholds are more stringent than those typically employed in previous MLP studies and highlight that adsorption enthalpies are particularly sensitive to errors in absolute energies. We further show that simple energy-offset corrections, combined with active-learning-based fine-tuning, provide an efficient strategy for achieving these accuracy targets.

The resulting MACE-AlMOFs-v1 model achieves DFT-level accuracy for 410 of the 420 Al-MOFs considered in this work, demonstrating that highly transferable machine-learning potentials can be constructed from a relatively small set of representative structures. Importantly, the model reproduces available experimental heats of adsorption and Henry coefficients significantly better than the commonly used UFF force field, which systematically underestimates water–framework interactions.

Beyond adsorption thermodynamics, our results reveal an additional, largely unexplored opportunity for machine-learning potentials in MOF research. Long-time scale MLP simulations can identify lower-energy framework configurations that conventional DFT geometry optimizations cannot access. For a large fraction of the Al-MOF data set, these alternative structures are substantially lower in energy than previously reported DFT-optimized configurations, underscoring the importance of enhanced configurational sampling when constructing MOF databases.

This work demonstrates that machine-learning potentials can bridge the gap between the accuracy of first-principles calculations and the scale required for high-throughput materials screening. The methodology developed here enables DFT-accurate screening of water adsorption in flexible MOFs and provides a general framework for extending foundation-model-based potentials to other porous materials and adsorption processes.

## Supplementary Material



## Data Availability

All structure files, DFT-labeled data sets, the optimal structures from MLP, and simulation results (i.e., *Q*_st_(H_2_O) and *K*_H_(H_2_O)) are available on Zenodo.^39^ The workflow for active learning and DFT calculations, along with the simulation codes, is available on GitHub at https://github.com/legend-L24/FlexSim.git.

## Notes

Further discussion about energy offsets; the extraction of Henry coefficients of CAU-10-X (X = H, NO_2_, and NH_2_) and *Q*_st_(H_2_O) of MIP-211; the relationship between the saturation uptakes and the accessible pore volume; the two MOFs where the MACE-MP-0b2 model fails to sample (PDF)
