# Supplementary material for: Generalized Machine Learning Potentials for Predicting Low-Pressure Water Adsorption in Flexible Al-Based Metal–Organic Frameworks
Source: J Chem Theory Comput. 2026 Aug 25;22(17):8976–89. doi: 10.1021/acs.jctc.6c01162 (PMC13564366; doi:10.1021/acs.jctc.6c01162)
Supplement: Supplementary file 1 [file ct6c01162_si_001.pdf]

Supporting Information: Generalized Machine Learning Potentials for Predicting Low-Pressure Water Adsorption in Flexible Al-Based Metal–Organic Frameworks

Yutao Li, Xiaoqi Zhang, Xin Jin, and Berend Smit*

Laboratory of molecular simulation (LSMO), Institut des Sciences et Ingénierie Chimiques, École Polytechnique Fédérale de Lausanne (EPFL), Rue de l'Industrie 17, CH-1951 Sion, Switzerland

E-mail: berend.smit@epfl.ch

Contents

S1 Systematic energy offsets **S-3**

S1.1 The influence of systematic energy offsets S-3

S1.2 Corrections for Energy Offsets S-6

S1.3 Model Validation on out-of-sample MOFs S-7

S2 Further discussion about accuracy threshold **S-12**

S2.1 Applicability of the threshold on new MOFs S-12

S2.2 Model transferability on different hydrogen bonds S-13

S3 Reweighted distances within SOAP descriptor space **S-14**

S4 Structures for which the Pretrained Model Failed **S-19**

S5 Analysis of the literature data **S-21**

S5.1 Extraction of Henry Coefficient S-21

S5.2 Estimation of Saturation Water Uptakes S-22

S5.3 Calculation of Zero-loading Heat Adsorption S-22

References **S-24**

S1 Systematic energy offsets

S1.1 The influence of systematic energy offsets

When we compute the heat of adsorption, we have to compute the difference between the average energy of the MOF with a water molecule and that of the pristine MOF.

$$Q_{\mathrm{st}} = -\langle E_{\mathrm{MOF+H_2O}} \rangle + \langle E_{\mathrm{MOF}} \rangle + \langle E_{\mathrm{H_2O}} \rangle + RT \tag{S1}$$

where $\langle E_{\mathrm{MOF+H_2O}} \rangle$, $\langle E_{\mathrm{MOF}} \rangle$, and $\langle E_{\mathrm{H_2O}} \rangle$ are the ensemble averages of the energy of the MOF with a water molecule, the pristine MOF, and a water molecule in the ideal gas phase, respectively.

If we use our machine-learning force field to, for example, optimize the structure, we do not have to worry about whether the absolute energy of our MOF is accurately described. We only need to predict the energy difference correctly. However, in equation (S1), we take the difference between two systems; hence, it is important that the same reference state be used for both systems. Hence, if we compare the difference in energy between our DFT reference and our MLP, this should be zero:

$$\Delta\Delta E^{\mathrm{Offset}} \equiv \Delta\bar{E}_{\mathrm{MOF}} - \Delta\bar{E}_{\mathrm{MOF+H_2O}} \tag{S2}$$

with

$$\Delta\bar{E}_{\mathrm{MOF+H_2O}} = \frac{1}{N}\sum_{i=1}^{N}\left[E_i^{\mathrm{DFT}}(\mathrm{MOF}+\mathrm{H_2O}) - E_i^{\mathrm{MACE}}(\mathrm{MOF}+\mathrm{H_2O})\right] \tag{S3}$$

$$\Delta\bar{E}_{\mathrm{MOF}} = \frac{1}{N}\sum_{i=1}^{N}\left[E_i^{\mathrm{DFT}}(\mathrm{MOF}) - E_i^{\mathrm{MACE}}(\mathrm{MOF})\right] \tag{S4}$$

where i runs over a set of configurations for which we evaluate both the DFT and the MACE force field. The impact of these offsets on the total energies of the heat of adsorption is given

by:

$$Q_{\text{st}} = Q_{\text{st}}^{\text{MACE}} + \Delta\Delta E^{\text{Offset}} \tag{S5}$$

One can expect that with an infinitely large training set, such offsets will become zero. However, as some of the training sets are infinite, it is important to quantify the extent of this effect. In Figure S1, we quantify these differences in the energy offsets for our set of Al-MOFs. In Figures S1a and S1b we report the difference in the total energy of our supercell predicted by the pretrained MACE-mp-0b2 model for which we carried out the DFT calculations. If this difference is just a constant that is the same for the MOF with water and the pristine MOF, a difference in the offsets of the total energy would be a non-issue. However, Figure S1c shows that the offset difference of the MOF with water and the pristine is not the same. For some MOFs, the difference exceeds the expected heats of adsorption. For this reason, using pretrained models out of the box to compute the heat of adsorption will yield poor results.

The other interesting observation is that the MACE-mp-0b2 model always overestimates potential energies for both pristine MOFs and MOFs with H_2O, as shown in Figures S1a and S1b. The MACE-mp-0b2 model is trained on the Materials Project trajectory dataset,[S1] which contains only inorganic crystal structures. As a result, the model does not learn bond dissociation energies among carbon atoms. While it may provide a reasonable description of atomic forces near equilibrium configurations, it lacks information on bond dissociation energies, such as C–C double bonds and aromatic rings. These bonding motifs, which stabilize the framework, are therefore ignored or underestimated by the pretrained model. To prove this inference, we plot the underestimation of potential energies by MACE-mp-0b2 (i.e., $-\Delta\bar{E}_{\text{MOF}}$) against the number of carbon atoms in pristine MOFs. As shown in Figure S1d, the degree of underestimation of potential energies increases systematically with the number of C atoms, supporting our explanation.

If we add training data, Figure S2 shows that the difference in total energies with the DFT results has decreased by two orders of magnitude. However, Figure S2c shows that

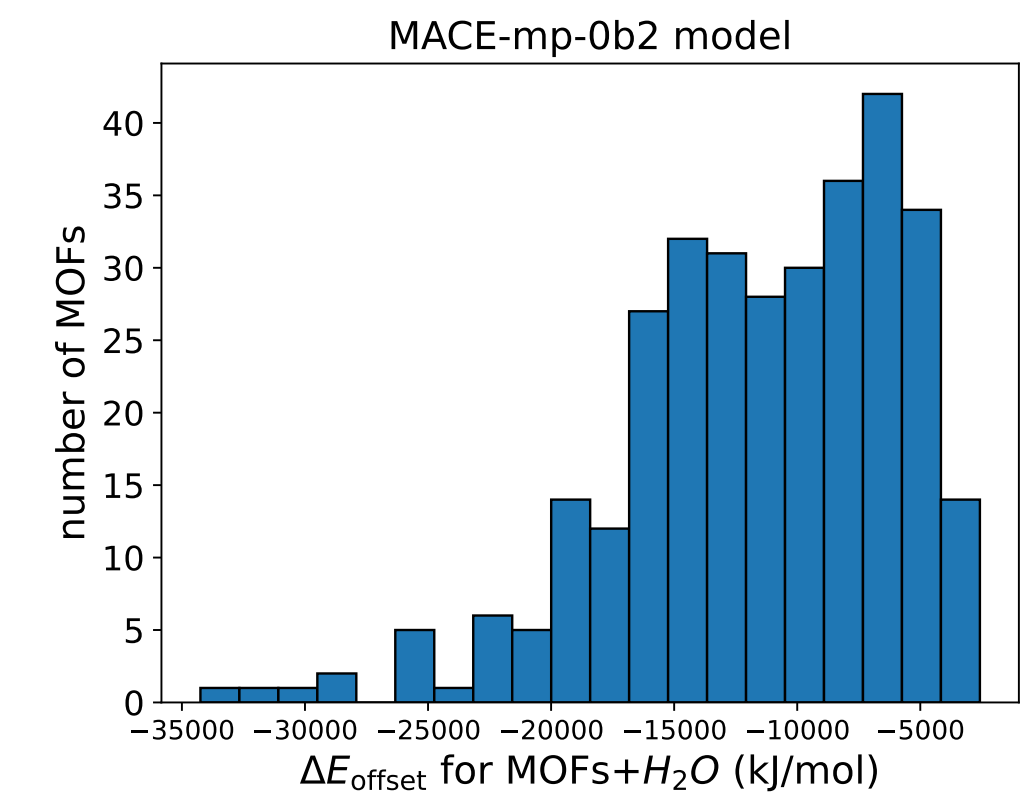

(a)

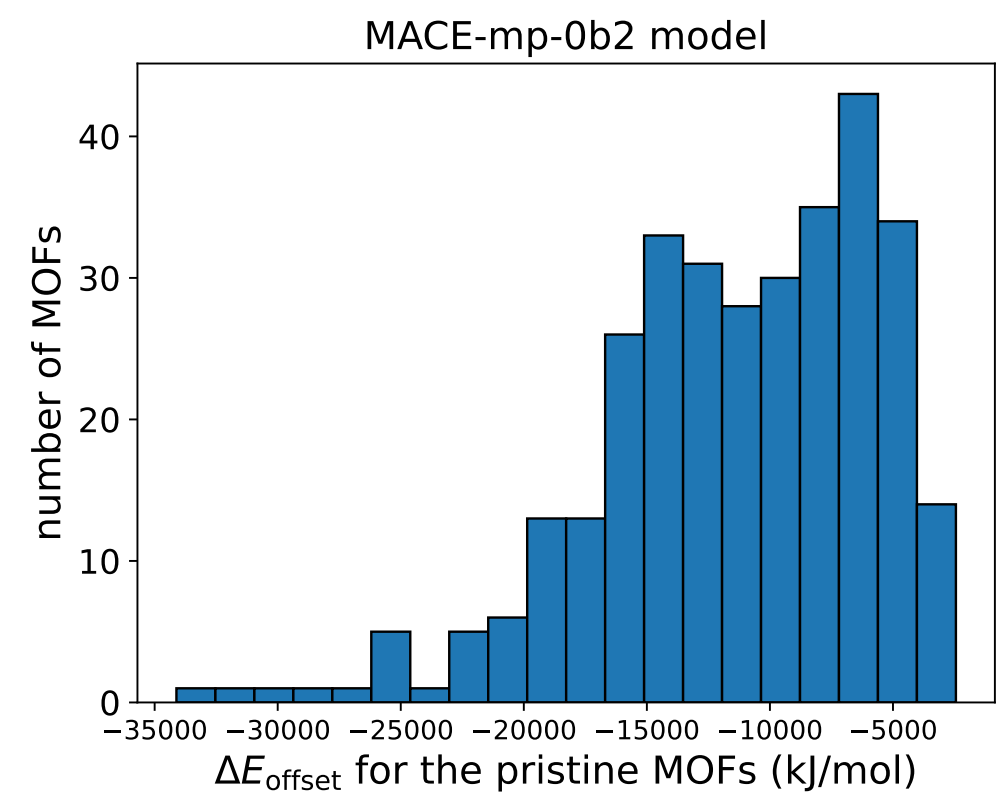

(b)

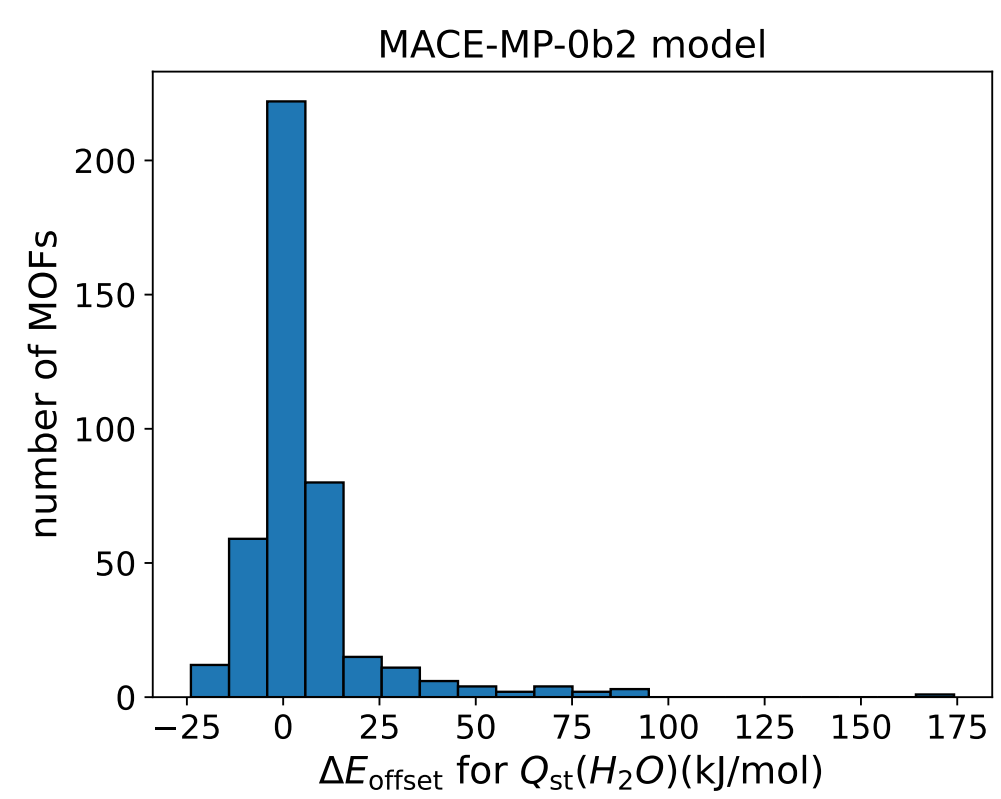

(c)

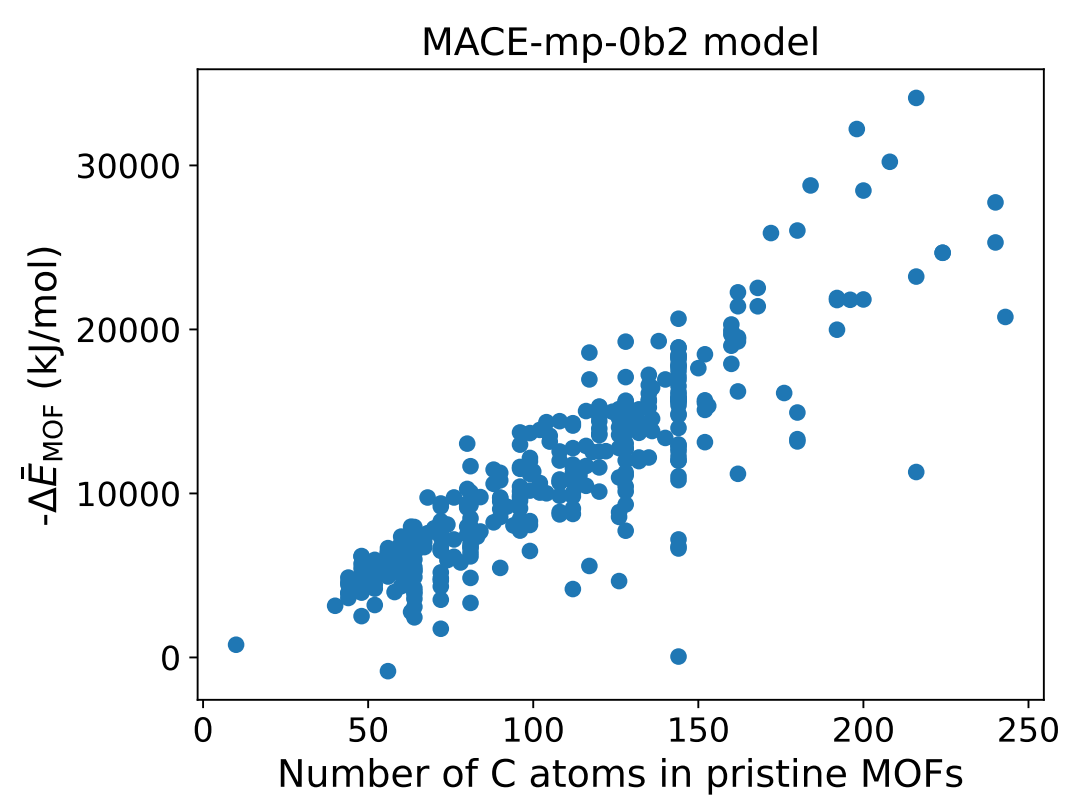

(d)

Figure S1: The energy offsets for 385 MOFs using the pretrained MACE-mp-0b model.[S2] (a) $\Delta\bar{E}_{\mathrm{MOF+H_2O}}$ (b) $\Delta\bar{E}_{\mathrm{MOF}}$ both in kJ per super cell (c) gives the impact of these shifts on the heat of adsorption ($\Delta\Delta E^{\mathrm{Offset}}$) in $\mathrm{kJ\,mol^{-1}}$ (d) The relationship between -$\Delta\bar{E}_{\mathrm{MOF}}$ and the number of C atoms in the supercell of pristine MOFs.

these differences are not zero, and for some MOFs it can give an error of 15.7 kJ mol^{-1}. As this can be 20% of the experimental heat of adsorption, it is important to correct for these differences in total energies. Since this correction is a simple constant, it can be added after the training, as it does not further impact the MLP.

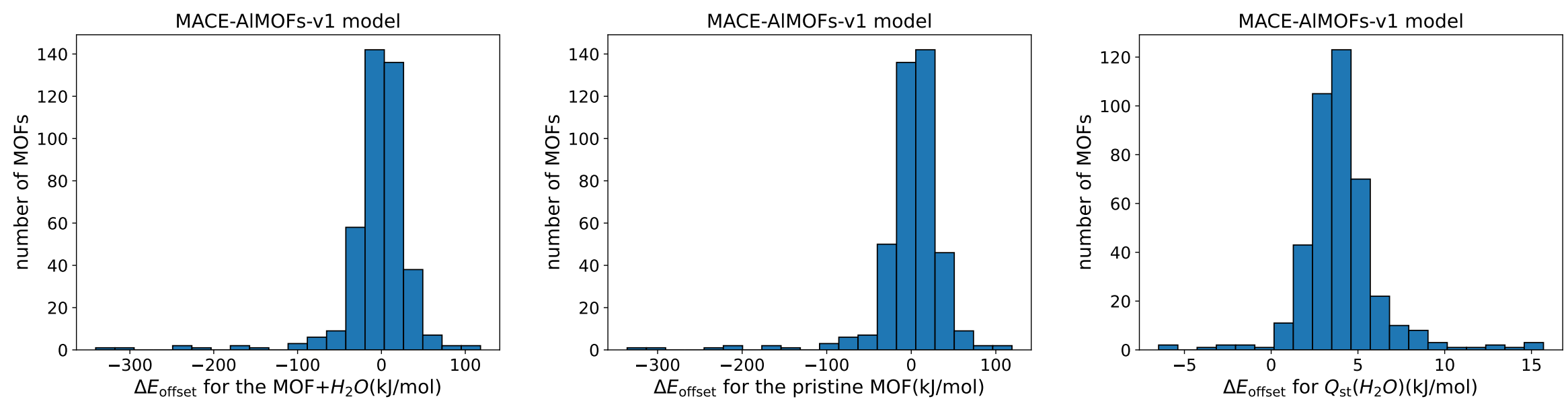

(a) **(b)** **(c)**

Figure S2: The energy offsets for 385 unique MOFs using the fine-tuned MACE model. The selected MOFs are the same to the ones in Figure S1 (a) $\Delta\bar{E}_{\mathrm{MOF+H_2O}}$ (b) $\Delta\bar{E}_{\mathrm{MOF}}$ both in kJ per super cell (c) gives the impact of these shifts on the heat of adsorption ($\Delta\Delta E^{\mathrm{Offset}}$) in kJ mol^{-1}.

S1.2 Corrections for Energy Offsets

We correct for the difference in total energy for a particular MOF using equations (S3) and (S4). For MOFs included in the training dataset, we can use them directly to evaluate the energy offsets. For each MOF, we obtain two constants ($\Delta\bar{E}_{\mathrm{MOF}}$ for pristine MOF and $\Delta\bar{E}_{\mathrm{MOF+H_2O}}$ for this MOF with a water molecule calculated by equation (S4)), and these MOF-dependent constants are then used to shift the predicted total energies. These shifts do not impact the forces.

Section S1.2 shows that correcting for total energies significantly decreases the mean-average errors (MAE) of the energies on the test dataset as compared to DFT. We observe a large improvement for those MOFs with large MAEs on potential energies. If the direct MAEs on potential energy are lower than 2 kJ mol^{-1}, we do not reduce the energy offsets, as we are approaching the accuracy of our training and the accuracy to determine the energy offset. With this correction, the remaining differences in offsets of total energies between the

MOF with water and the pristine MOF have a negligible effect on the heats of adsorption.

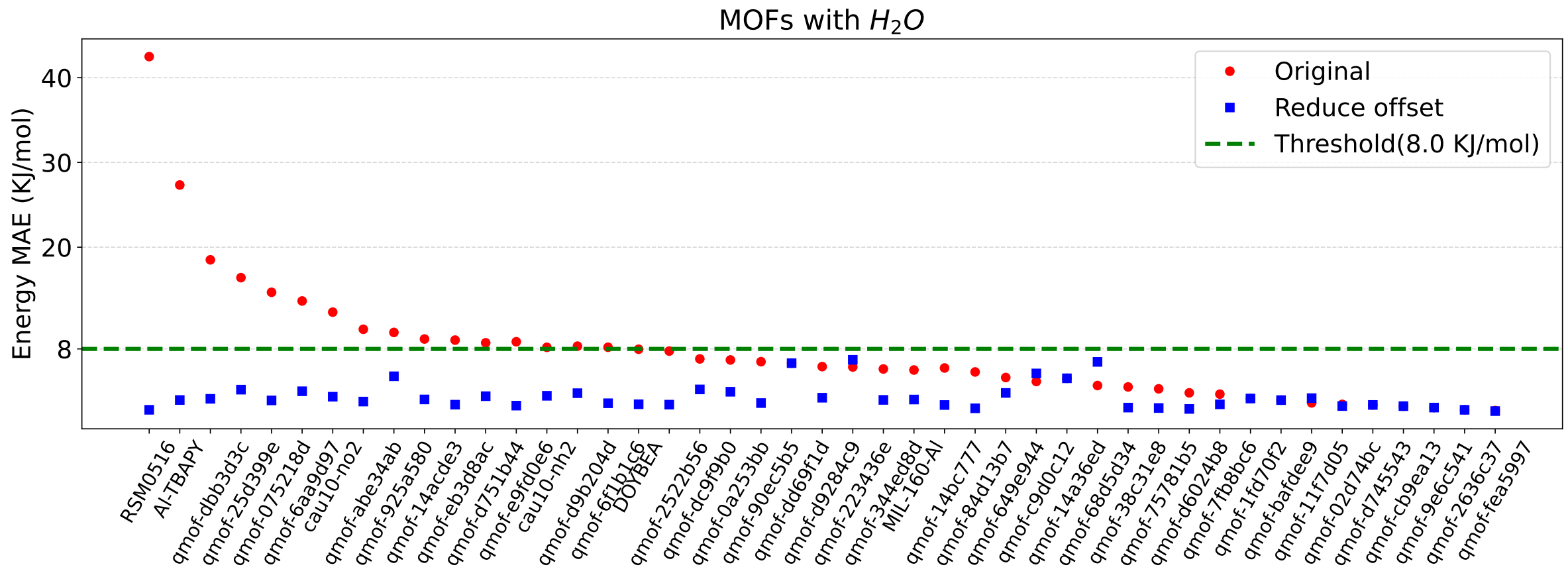

(a)

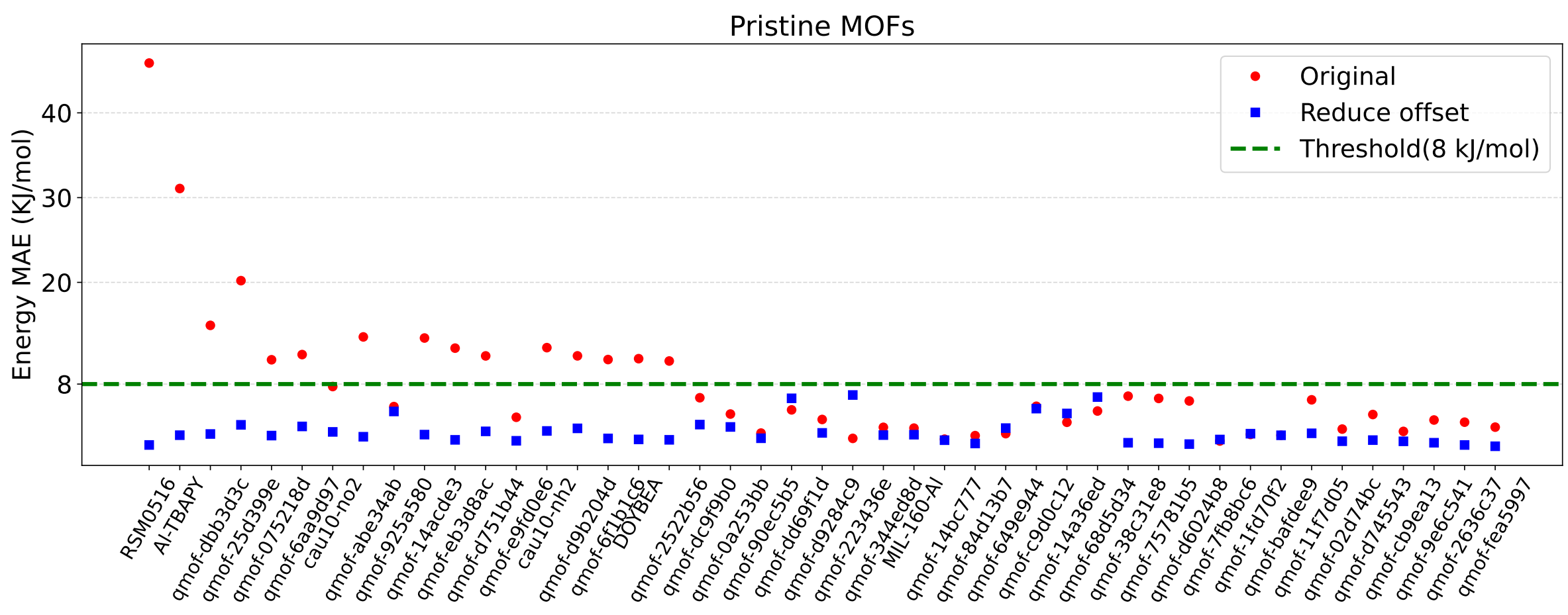

(b)

Figure S3: (a) The MAEs on potential energies for the MACE-AlMOFs-68 model on the test dataset including 45 pristine MOFs with H_2O. These MOFs are included in the training dataset. The red points represent the original MAEs, and the blue points represent those after reducing energy offsets. (b) The MAEs on potential energies for the MACE-AlMOFs-68 model on the test dataset, including 45 pristine MOFs. The red points represent the original MAEs, while the blue points show those after reducing energy offsets.

S1.3 Model Validation on out-of-sample MOFs

We have trained our MACE-AlMOFs-68 model using data of 68 representative Al-MOFs with a diverse set of linkers. Hence, we expect this model to give a much better description than the original MACE model for similar Al-containing MOFs.

In this section, we provide guidance on applying our MACE-AlMOFs-68 model to an

MOF not included in the training set. We have used this procedure successfully for 352 MOFs in the main text; therefore, this section focuses on Al-MOFs that are not part of these 352.

Of course, the MACE-AlMOFs-68 model can be used out of the box. For 352 out-of-sample MOFs, we have a 79% chance that no additional fine-tuning is needed to have a sufficiently accurate model to carry out an MD simulation of the pristine MOF or MOF with H_2O at 300 K. (i.e., RMSEs on atomic forces lower than 50 meV Å^{-1}) Recall that our criteria are much stricter than those typically used in the literature. Indeed, we can use this force field to carry out an energy minimization and determine some properties of the MOF (e.g., thermal conductivity, mechanical strength).

However, if we want to use these simulations to compute the heat of adsorption, or any other simulation that requires computing the difference in energy between two different systems, we also need to have the energy predicted sufficiently accurately. To illustrate the role of corrections in total energies, we randomly select 40 MOFs from 352 out-of-sample MOFs and represent energy and force errors for each pristine MOF in Figures S4a and S4b. Figure S4a shows that the errors on potential energies are, for most MOFs, much higher than our threshold, mainly due to a systematic offset in the total energies. For the correction in total energy, we sample 60 configurations (N) taken from an MD trajectory, which are independent of the test dataset. After reducing energy offsets, the MACE-AlMOFs-68 model satisfies our strict criteria on 77% of MOFs, in which the model can provide reliable predictions of $Q_{\mathrm{st}}(H_2O)$. In addition, it is worth noting that the remaining energy errors after energy correction are related to large force errors. As shown in Figure S4b, for those MOFs with large force errors, the energy errors after reducing offsets are still higher than our threshold.

To further illustrate the influence of energy offsets on $Q_{\mathrm{st}}(H_2O)$, we estimate the errors on $Q_{\mathrm{st}}(H_2O)$ of 352 out-of-sample MOFs using the test dataset of the pristine MOFs and the MOFs with H_2O. We split these MOFs into two subsets: (a) MOFs with RMSEs on

atomic forces lower than 50 meV Å^{-1}; (b) MOFs with RMSEs(force) higher than 50 meV Å^{-1}. Because RMSEs lower than 50 meV Å^{-1} can ensure that the model can provide correct MD samplings, we are more concerned about the errors for the first subsets. As shown in Figure S5a, for 279 MOFs in the first subset, an error of about 15 kJ mol^{-1} can be expected for $Q_{st}(H_2O)$, and the correction in total energies can significantly improve it. For 73 MOFs in the second subset, an error of about 40 kJ mol^{-1} can be expected for $Q_{st}(H_2O)$.

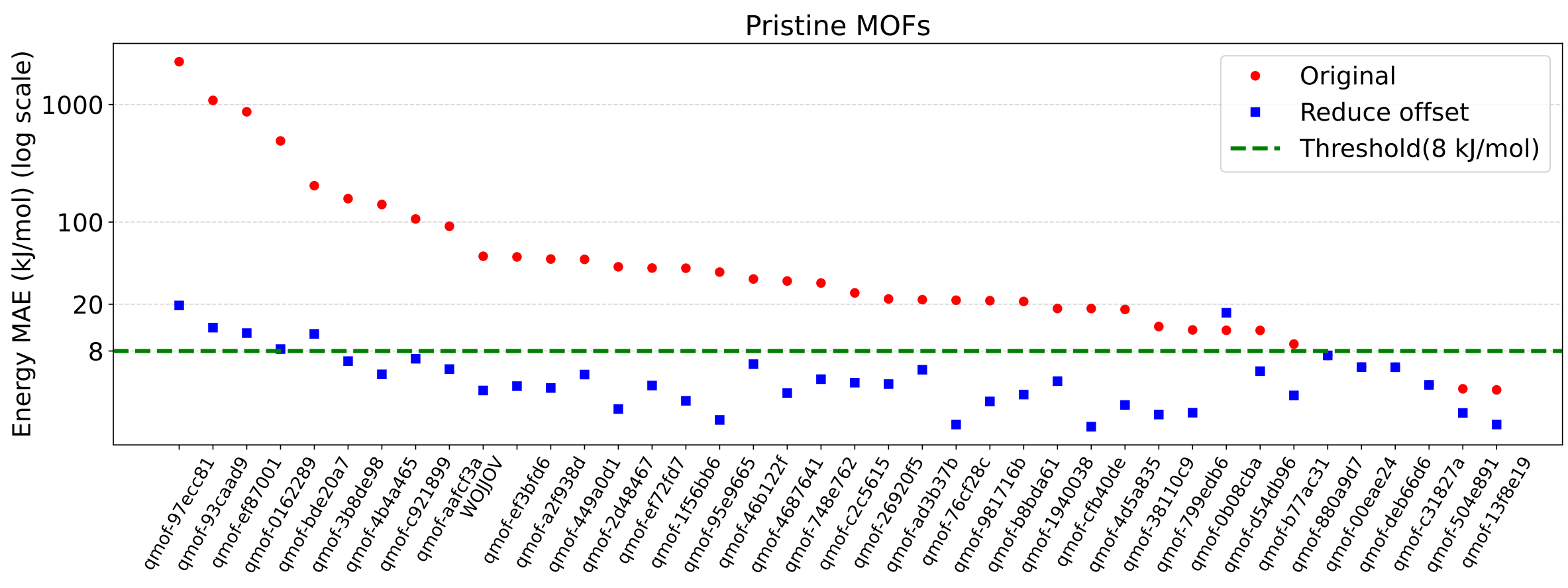

(a)

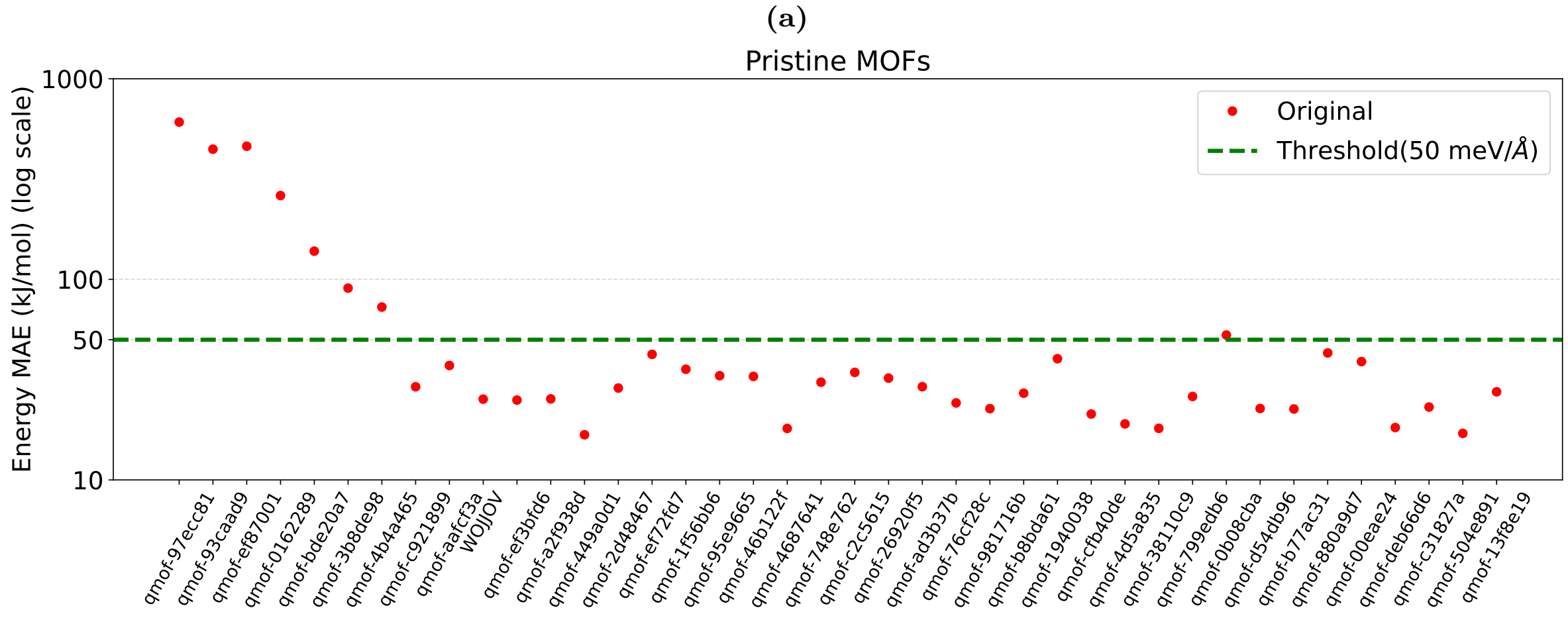

(b)

Figure S4: The errors of MACE-AlMOFs-68 models on out-of-sample MOFs. We randomly select 40 MOFs from 352 out-of-sample MOFs and plot their errors separately. All errors for the pristine MOFs and MOFs with H_2O can be found in the main text. (a) The MAEs on potential energies for the MACE-AlMOFs-68 model on the test dataset, including 45 pristine MOFs. The red points represent the original MAEs, and the blue points represent those with reduced energy offsets. (b) The RMSEs on atomic forces on the same test dataset, including 45 pristine MOFs.

It is relatively straightforward to correct for these offsets in total energies. We use the pristine qmof-5ab6b28 structure as an example, in which the MACE-AlMOFs-68 model has

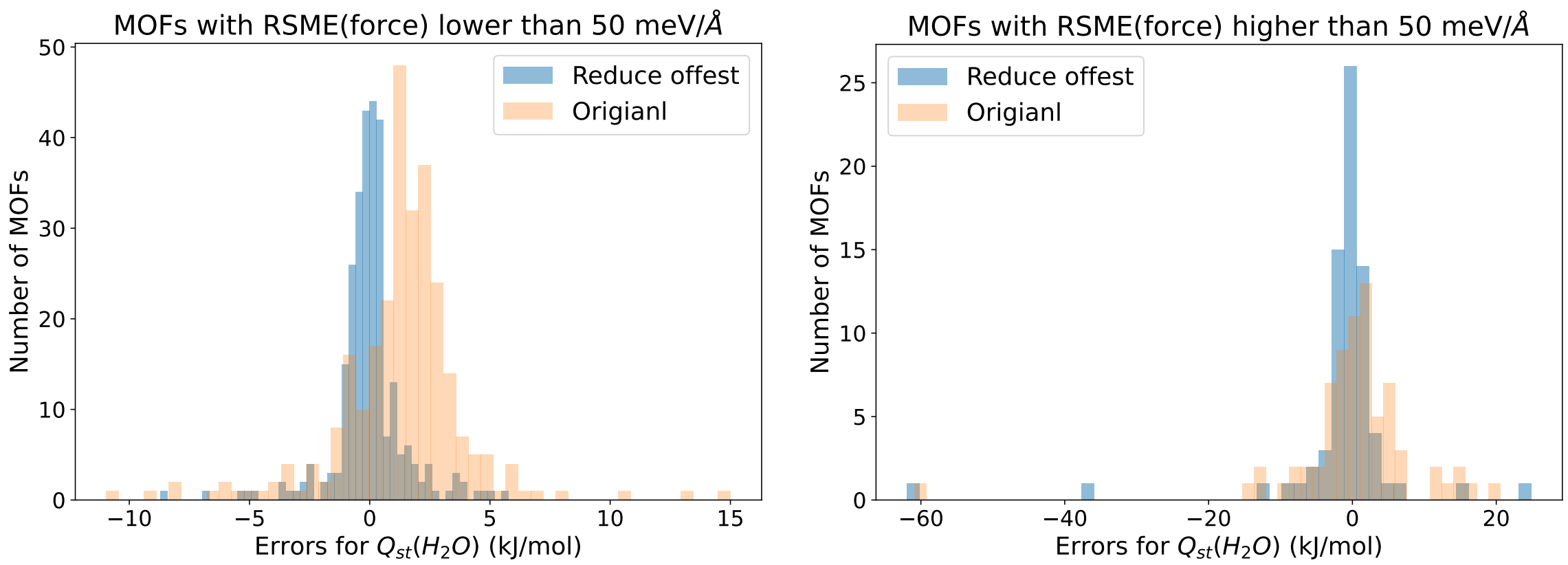

(a) **(b)**

Figure S5: Histograms of errors in $Q_{\mathrm{st}}(\mathrm{H_2O})$ (i.e., $\Delta\bar{E}_{\mathrm{MOF}}$ - $\Delta\bar{E}_{\mathrm{MOF+H_2}O}$) predicted by the MACE-AlMOFs-68 model for 352 out-of-sample MOFs. The orange bars show errors on directly predicted $Q_{\mathrm{st}}(\mathrm{H_2O})$, and the blue ones are after subtracting energy offsets. These errors are estimated on the test dataset, and the determination of energy offsets is on the additional DFT-labeled configurations outside the test dataset. (a) Subset of MOFs with RMSE on atomic forces lower than 50 meV Å^{-1}, where the model provides reliable MD trajectories. (b) Subset of MOFs with larger force errors.

49 meV Å^{-1} of RMSEs on atomic forces. The energy errors (i.e., $E_{DFT} - E_{MACE}$) on the test dataset of the pristine MOF are shown in Figure S6a and the correction term can decrease the MAE on potential energies from 2.1 meV/atom to 0.25 meV/atom.

Figure S6b shows how the value of the energy offsets depends on the number of DFT calculations for a particular MOF. In principle, we only need to carry out one calculation to compute this shift. However, given the uncertainties in the force field, a more accurate result is obtained by using 10 DFT calculations. As shown in Figure S6b, only ten DFT calculations are enough to estimate the energy offset for pristine qmof-5ab6b28 accurately. Furthermore, if we apply only 10 DFT calculations to estimate the energy offsets of 352 out-of-sample MOFs, the MACE-AlMOFs-68 model remains transferable to 267 unique MOFs. and these MOFs with H_2O. By comparison, the fine-tuned model meets our criteria for 271 MOFs when the full dataset (i.e., 60 configurations per system) is used to evaluate energy offsets. Therefore, the correction in total energies provides a simple and efficient way to improve energy accuracy for out-of-sample MOFs, particularly those exhibiting low force errors. (i.e, RMSEs on atomic forces lower than 50 meV Å^{-1})

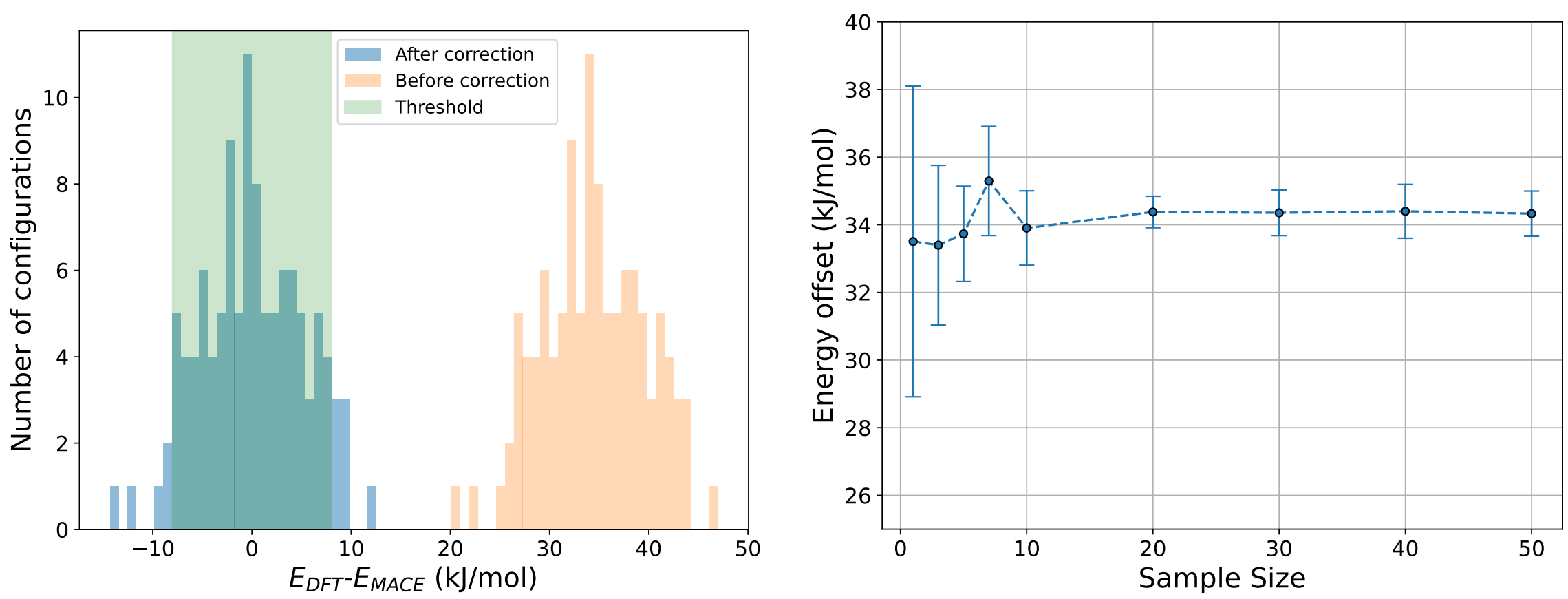

(a) **(b)**

Figure S6: (a) Histogram of energy errors ($E_{DFT} - E_{MACE}$) for 112 pristine qmof-5ab6b28 configurations. (b) For each sample size, we randomly select 8 subsets of that size and calculate the energy offsets. The relationship between the averages and uncertainties of these offsets and sample sizes is shown here.

S2 Further discussion about accuracy threshold

S2.1 Applicability of the threshold on new MOFs

In the main text, we use water adsorption in MIL-160 to establish energy and force error thresholds of 10 kJ mol^{-1} per configuration and 40 meV Å^{-1}, respectively, for reliable predictions of Q_{st}. To examine whether these thresholds apply to chemically distinct MOFs, we performed similar tests for MFM-300 and KMF-1.[S3,S4]

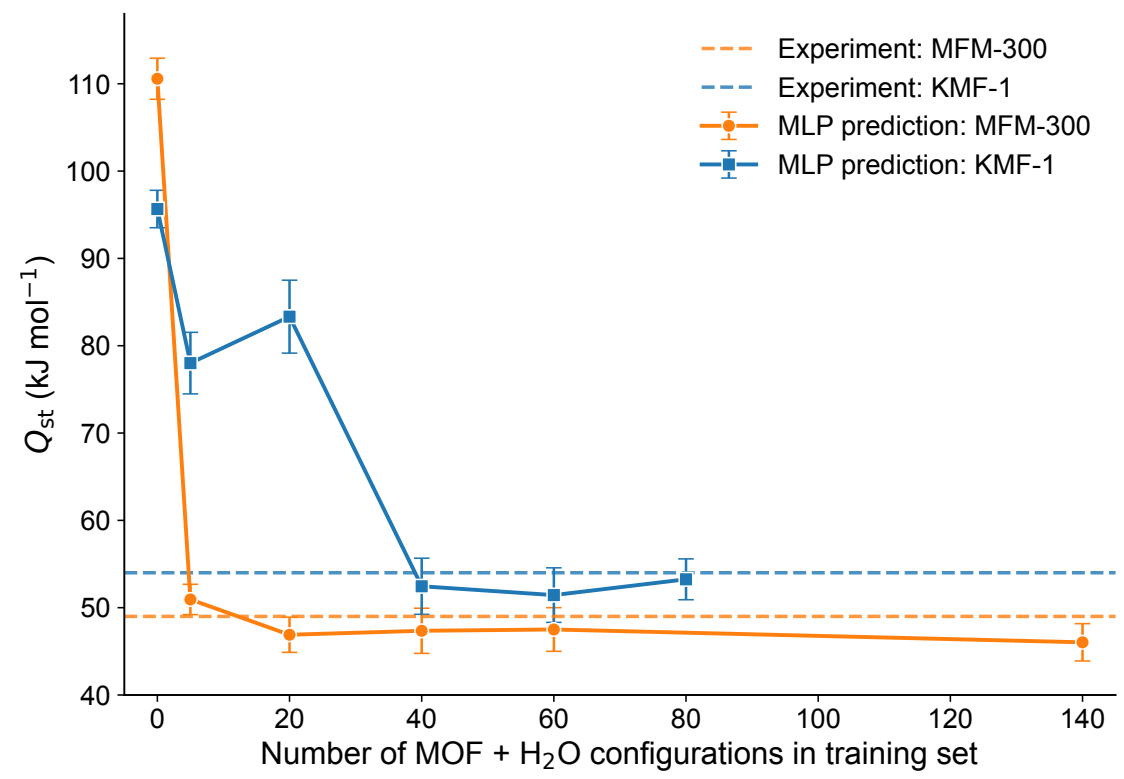

Figure S7: Predicted isosteric heat of adsorption, $Q_{st}(H_2O)$, for MFM-300 and KMF-1 as a function of the number of DFT-labeled configurations of MOF with H_2O used to fine-tune the MACE model. Each point stands for a fine-tuned model. In their training set, we kept the same number of pristine MOF and isolated H_2O configurations, but added a different number of configurations of MOF with H_2O. Error bars are the deviation of simulated $Q_{st}(H_2O)$ from multiple MD simulations using the same fine-tuned model with the time scale of 20 ps.[S3,S4]

For each MOF, we fine-tuned six MACE models using training datasets containing the same, sufficiently large numbers of pristine-MOF and isolated-H_2O configurations but different numbers of MOF+H_2O configurations. The resulting models therefore exhibit different errors for the MOF+H_2O systems and were used to calculate $Q_{st}(H_2O)$. As shown in Figure S7, increasing the number of MOF+H_2O training configurations improves the model accuracy and causes the predicted $Q_{st}(H_2O)$ to converge toward the experimental values. The corresponding energy and force errors are shown in Figure S8.

As shown in Figure S8b, the force errors already satisfy either the threshold adopted in this work or that reported previously.[S5] The energy error is therefore the main limiting factor and requires additional training data to converge. Consequently, the four least accurate

models in Figure S7 overestimate Q_{st} by more than $25\,kJ\,mol^{-1}$. When the force accuracy is already sufficient, the remaining large energy errors can be substantially reduced by applying the energy-offset correction described in Section S1. Overall, for both MOFs, models satisfying our energy and force error thresholds reproduce the experimental Q_{st} values. These results support the applicability of the proposed thresholds to chemically diverse MOFs.

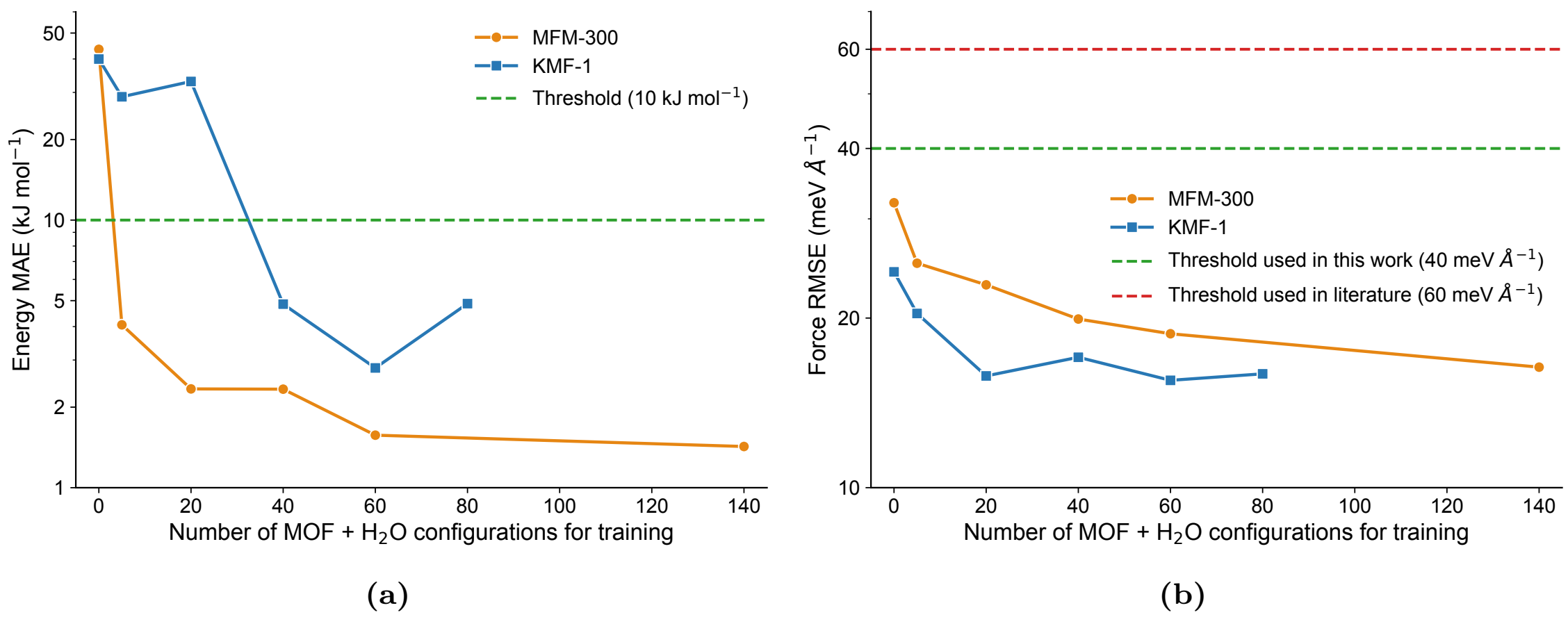

Figure S8: (a) Mean average errors (MAEs) on potential energies ($kJ\,mol^{-1}$ per configuration) of the test dataset of MFM-300 with H_2O and KMF-1 with H_2O as a function of the number of MOF with H_2O configurations in the training dataset. (b) Root Mean Square Errors (RMSEs) on atomic forces of the test dataset of MFM-300 with H_2O and KMF-1 with H_2O as a function of the number of MOF with H_2O configurations in the training dataset. The green lines indicate the error thresholds below which the MLP yields reliable predictions of Q_{st}. The red one is the threshold used in previous work[S5]

S2.2 Model transferability on different hydrogen bonds

In the main text, we fine-tuned the MACE model using different training datasets and calculated $Q_{st}(H_2O)$ for MIL-160 to reveal the relationship between model errors and reliable $Q_{st}(H_2O)$ calculations. These training datasets all contain sufficient configurations of pristine MIL-160 and isolated H_2O molecules (water in the gas phase), but different numbers of configurations of MIL-160 with H_2O. In addition, we added liquid-water configurations (16 H_2O molecules in a unit cell at the density of liquid water) to these training datasets and fine-tuned the models again for a similar test.

There is a remarkable observation. When no configurations of water in the MOF are

included in the training dataset, we observe a large difference between the predictions represented by the blue and orange curves in Figure S9a. The difference is that the models represented by the orange curve are trained on water configurations in both the gas and liquid phases, whereas those represented by the blue curve are trained only on gas-phase water configurations. Therefore, including liquid-water configurations greatly improves the predictions and even yields $Q_{st}(H_2O)$ values consistent with the experimental values without including any configurations of MIL-160 with H_2O in the training dataset. Figure S9b also shows that liquid-water data reduce energy errors for MIL-160 with H_2O when only a limited number of these configurations are included in the training dataset. This improvement may arise because hydrogen bonds between H_2O and the μ_2-OH groups make a significant contribution to the heat of adsorption. These results suggest that the MACE model can transfer knowledge learned from hydrogen bonds between H_2O molecules to those between H_2O and the MOF, providing insight into the transferability of machine-learning potentials. Interestingly, comparable accuracy and correct $Q_{st}(H_2O)$ predictions can also be achieved by the models trained only on gas-phase water, but substantially more configurations of MIL-160 with H_2O are required in the training dataset, as shown in Figures S9a and S9b.

S3 Reweighted distances within SOAP descriptor space

The transferability indicator introduced in the main text is based on the hypothesis that the accuracy of the MACE-AlMOFs-68 model depends on how well the local chemical environments of a new MOF are represented in the training dataset. To quantify this relationship, we first define a similarity metric based on distances between this new MOF and the training dataset in SOAP descriptor space and subsequently introduce a learned reweighting scheme that improves its correlation with force prediction errors.

For each atom i in an out-of-sample MOF, its local chemical environment is represented by a SOAP descriptor $\mathbf{s}_i$. We identify the most similar environment in the training dataset

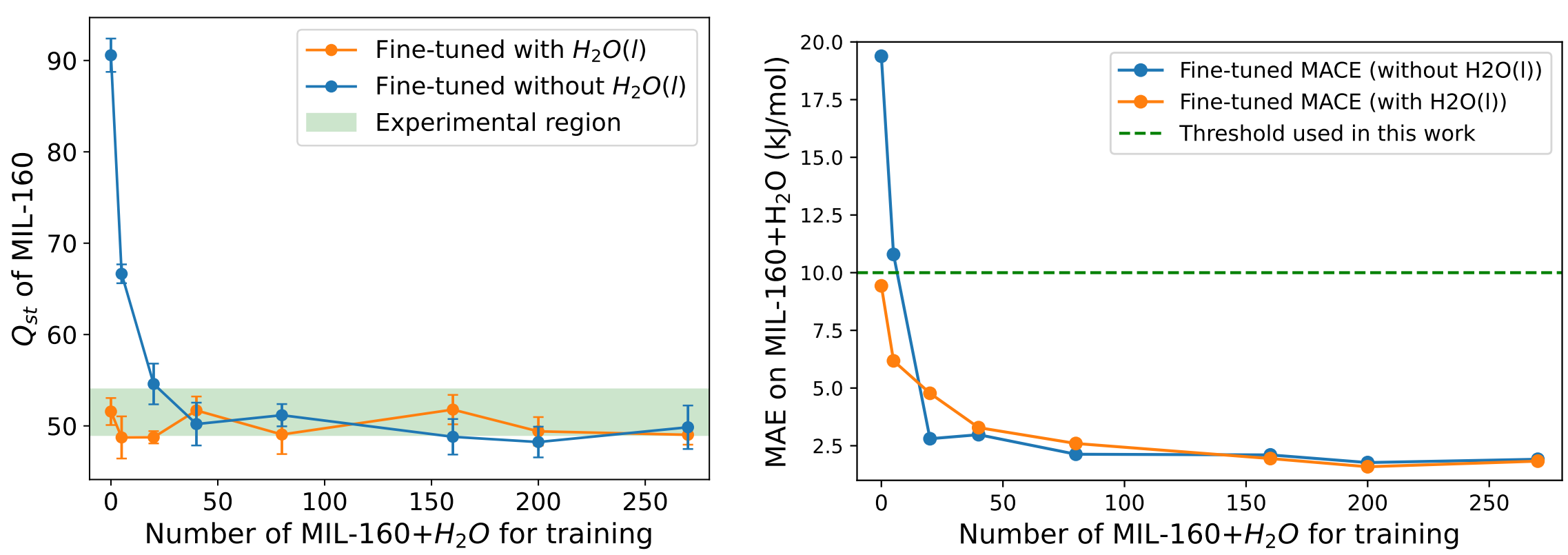

(a) **(b)**

Figure S9: (a) Predicted isosteric heat of adsorption, $Q_{\mathrm{st}}(\mathrm{H_2O})$, for MIL-160 as a function of the number of MIL-160 with H_2O configurations in the training dataset. Each point stands for a fine-tuned model. The training datasets of these models also contain sufficient configurations of the pristine MIL-160 and water molecules without the MOF. The difference between the orange curve and the blue one is that the models in the orange line are fine-tuned using data from both the gas and liquid phases of water, whereas those of the blue line are fine-tuned with only gas-phase data. Error bars are the deviation of simulated $Q_{\mathrm{st}}(\mathrm{H_2O})$ from multiple MD simulations using the same fine-tuned model with the time scale of 20 ps. The green band is the range in which the experimental heat of adsorption is reported.[S6–S8] (b) Mean average errors (MAEs) on potential energies of the test dataset of MIL-160 with H_2O as a function of the number of MIL-160 with H_2O configurations in the training dataset. The difference between the orange curve and the blue one is also that the training dataset includes liquid-phase water data for models in the orange curve.

by calculating the minimum cosine distance,

$$d_i = \min_{j \in \mathcal{D}_{\text{train}}} d_{\cos}\left(\mathbf{s}_i, \mathbf{s}_j\right), \tag{S6}$$

where $\mathcal{D}_{\text{train}}$ denotes the collection of all atomic environments contained in the MACE-AlMOFs-68 training dataset. The similarity between an entire MOF and the training set is then quantified as the root-mean-square of these minimum distances because we compared this metric with RMSEs on atomic forces,

$$D_{\text{SOAP}} = \sqrt{\frac{1}{N}\sum_{i=1}^{N} d_i^2}, \tag{S7}$$

where N is the number of atoms in the MOF.

Figure S10a shows the distribution of D_{SOAP} for transferable and non-transferable MOFs. Transferable MOFs generally exhibit smaller SOAP distances, indicating that model transferability is related to the coverage of local environments in the training dataset. However, the separation between the two classes remains incomplete. This observation suggests that geometric distances alone are insufficient for predicting force accuracy. A distance metric only characterizes the magnitude of the displacement in SOAP descriptor space, but does not specify the region of descriptor space in which that displacement occurs. Consequently, identical SOAP distances may correspond to substantially different force errors. This behavior is expected because the force landscape is highly heterogeneous: local environments associated with strongly varying interactions are considerably more sensitive to descriptor-space perturbations than environments located in smoother regions. Therefore, transferability cannot be determined solely from the distance to the nearest training environment; the local chemical environment itself must also be taken into account.

To incorporate this information, we introduce an environment-dependent weighting function. A three-layer neural network is trained to predict a scalar weight w_i from the SOAP descriptor of each atomic environment of A new MOF. The weighted distance for atom i is

defined as

$$d_i^{\mathrm{w}} = w_i d_i. \tag{S8}$$

The weighted distances are used to estimate the force error associated with each atomic environment,

$$\mathrm{RMSE}_{\mathrm{atom},i}^{\mathrm{pred}} = w_i d_i. \tag{S9}$$

The neural network parameters are optimized by minimizing the difference between the predicted and actual atomic force errors,

$$\mathcal{L} = \sqrt{\frac{1}{N_{\mathrm{atom}}} \sum_{i=1}^{N_{\mathrm{atom}}} \left(\mathrm{RMSE}_{\mathrm{atom},i}^{\mathrm{pred}} - \mathrm{RMSE}_{\mathrm{atom},i}^{\mathrm{true}}\right)^2}, \tag{S10}$$

where N_{atom} denotes the total number of atomic environments included in the training set of the transferability indicator model, including 176 unique MOFs. After training, the predicted atomic force errors are aggregated to obtain a MOF-level transferability metric,

$$\mathrm{RMSE}_{\mathrm{MOF}}^{\mathrm{pred}} = \sqrt{\frac{1}{N} \sum_{i=1}^{N} \left(\mathrm{RMSE}_{\mathrm{atom},i}^{\mathrm{pred}}\right)^2}. \tag{S11}$$

The 352 out-of-sample MOFs were randomly divided into equal training and test subsets containing 176 MOFs each. Using Section S3, the predicted atomic force errors are aggregated into an MOF-level force RMSE, which serves as a transferability indicator for unseen MOFs. The predicted RMSEs correlate strongly with the actual force errors evaluated using DFT-labeled configurations ($R^2 = 0.90$), indicating that the weighted SOAP distance provides a reliable estimate of model transferability.

As shown in Figure S10b, the weighted SOAP distance provides substantially improved separation between transferable and non-transferable MOFs. Transferable MOFs are concentrated at small weighted distances, whereas non-transferable MOFs are shifted toward

larger values. Using a threshold of 0.04, transferable and non-transferable MOFs in the test dataset can be identified with accuracies of 85% and 93%, respectively. These results demonstrate that the learned reweighting scheme converts a purely geometric similarity metric into a transferability-aware distance metric that can be used to estimate the reliability of the MACE-AlMOFs-68 model for unseen MOFs.

Beyond transferability assessment, the learned weighting function also provides insight into dataset construction. Environments assigned larger weights correspond to regions of SOAP descriptor space where force predictions are particularly sensitive to local structural variations and therefore require denser sampling. The weighted SOAP distance may therefore serve as a useful guide for future diversity-based sampling and active-learning strategies aimed at expanding the transferability of machine-learning potentials.

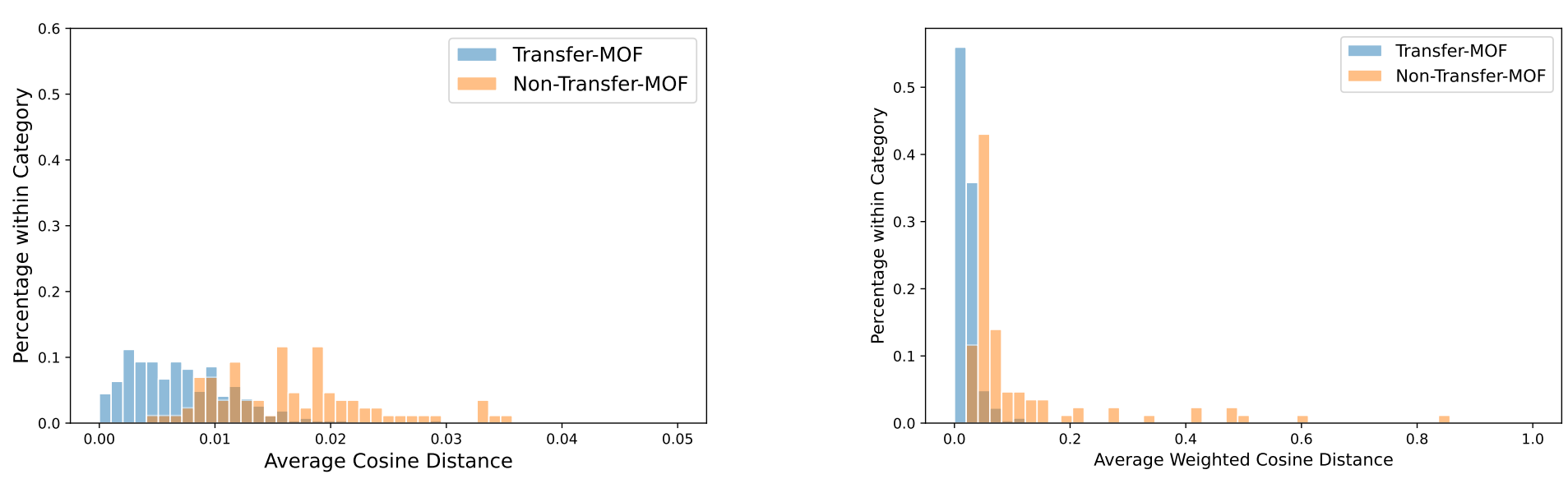

(a) (b)

Figure S10: Distribution of distances between out-of-sample MOFs and the MACE-AlMOFs-68 training dataset. (a) Distribution of minimum cosine distances in the original SOAP descriptor space. (b) Distribution of distances in the weighted SOAP descriptor space, where environment-dependent weights are learned from SOAP descriptors to reproduce force prediction errors. Blue and orange correspond to transferable and non-transferable MOFs, respectively, classified using a force RMSE threshold of $40\,\mathrm{meV}\,\text{Å}^{-1}$. Reweighting substantially improves the separation between the two classes, enabling transferability prediction using a simple distance threshold.

S4 Structures for which the Pretrained Model Failed

Compared with training a machine learning potential (MLP) from scratch, fine-tuning a pretrained model can reduce the amount of training data required to reach the same level of accuracy.[S9] Beyond this data efficiency, the pretrained model offers another key advantage: molecular dynamics (MD) stability. In practice, this means that pretrained models do not generate nonphysical structures during long MD simulations. At the initialization stage, a pretrained model can be directly employed for sampling, which can avoid the need for expensive ab initio MD to bootstrap the training.[S10] During the early stages of active learning, the fine-tuned model inherits the MD stability of the pretrained model and can thus be reliably used for long MD sampling, such as 50 ps used in this paper. In contrast, MLPs trained from scratch often exhibit fragile MD stability, restricting simulations to short timescales (e.g., 1 ps[S11]). Longer MD simulations allow a more comprehensive exploration of configuration space, which in turn reduces the required number of active-learning cycles.

Our work also benefits from the MD stability of the pretrained model. In the initialization stage, we employ the MACE-mp-0b2 model to sample the 70 representative MOFs and their water loading configurations. This pretrained model shows good MD stability on most MOFs, except for two MOFs: qmof-bdc796 and qmof-451bd4c.

In the first step of our training, we select 100 configurations from the MD trajectories. For qmof-bdc796 and qmof-451bd4c, we find that 10% of the structures are problematic, i.e., we cannot converge the energy in the DFT calculation. Both MOFs consist of one-dimensional infinite Al-O chains and ligands with two carbonyl groups. The ligands link the one-dimensional Al–O chains into a framework featuring parallelogram-shaped channels. In Figure S11, we show the metal clusters and ligands of both MOFs before and after the NVT simulation by the MACE-mp-0b2 model. In this simulation, the metal clusters are stable while the ligands generate nonphysical configurations. The double bond between carbon atoms is broken, and $sp\hat{2}$-C atoms convert to $sp\hat{3}$-C atoms, which generate a five-member hetero-ring. Although each atom adopts a seemingly reasonable local bonding configuration,

the resulting rings are unstable because free radicals are produced. From these two examples, we found two shortcomings of the MACE-mp-0b2 model: (1) the pretrained model can not identify some unstable configurations. (2) The pretrained model underestimates the energy barrier of chemical bond breaking. These shortcomings come from the training dataset of the MACE-mp-0b2 model, which only includes the structure relaxation and static calculation of inorganic crystals. Similar problems are also found in other works. Zhang et al.[S12] also finds that the paddle-wheel zinc metal clusters are dissociated into two clusters in the NVT simulation at 300 K of the MACE-mp-0b2 model.

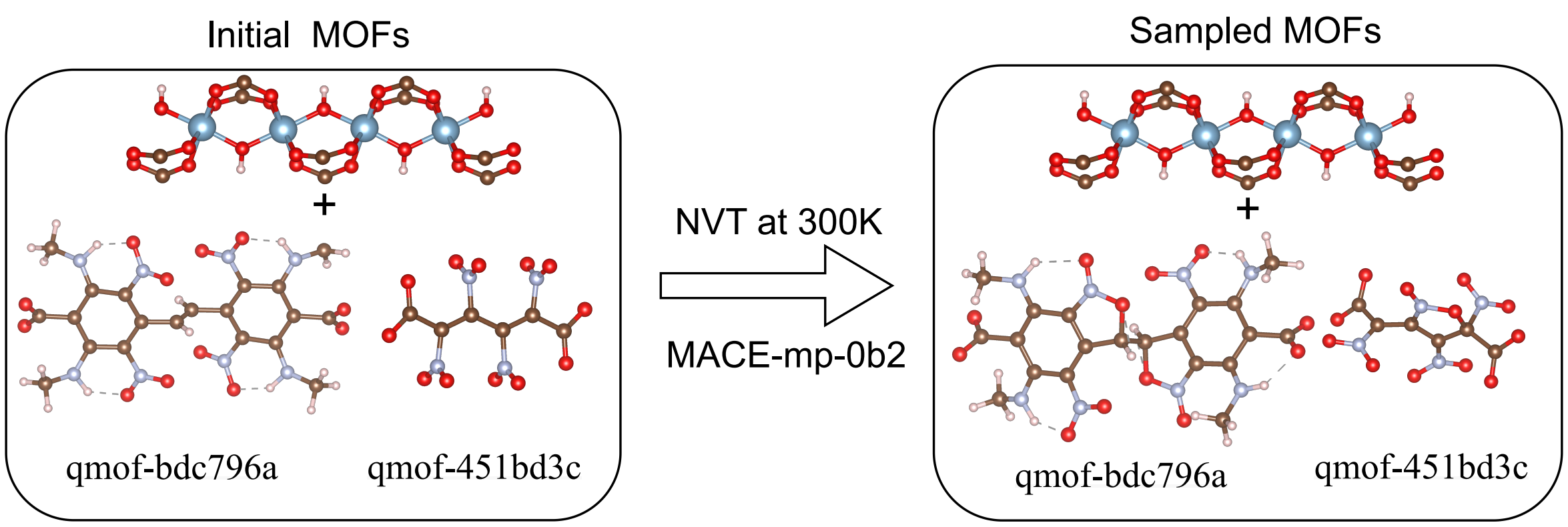

Figure S11: The metal clusters and ligands of the two MOFs: qmof-bdc796 and qmof-451bd4c. The left one is the initial configuration and the right one is the snapshot from the MD trajectory sampled by the MACE-mp-0b2 model.

S5 Analysis of the literature data

S5.1 Extraction of Henry Coefficient

The experimental Henry coefficients are extracted from low-pressure experimental isotherms of CAU-10-X (X=H, NO_2, NH_2).[S13] The linear regression figures are shown in Figure S12a, S12b, and S12c, where the coefficients of determination R^2 are larger than 0.98.

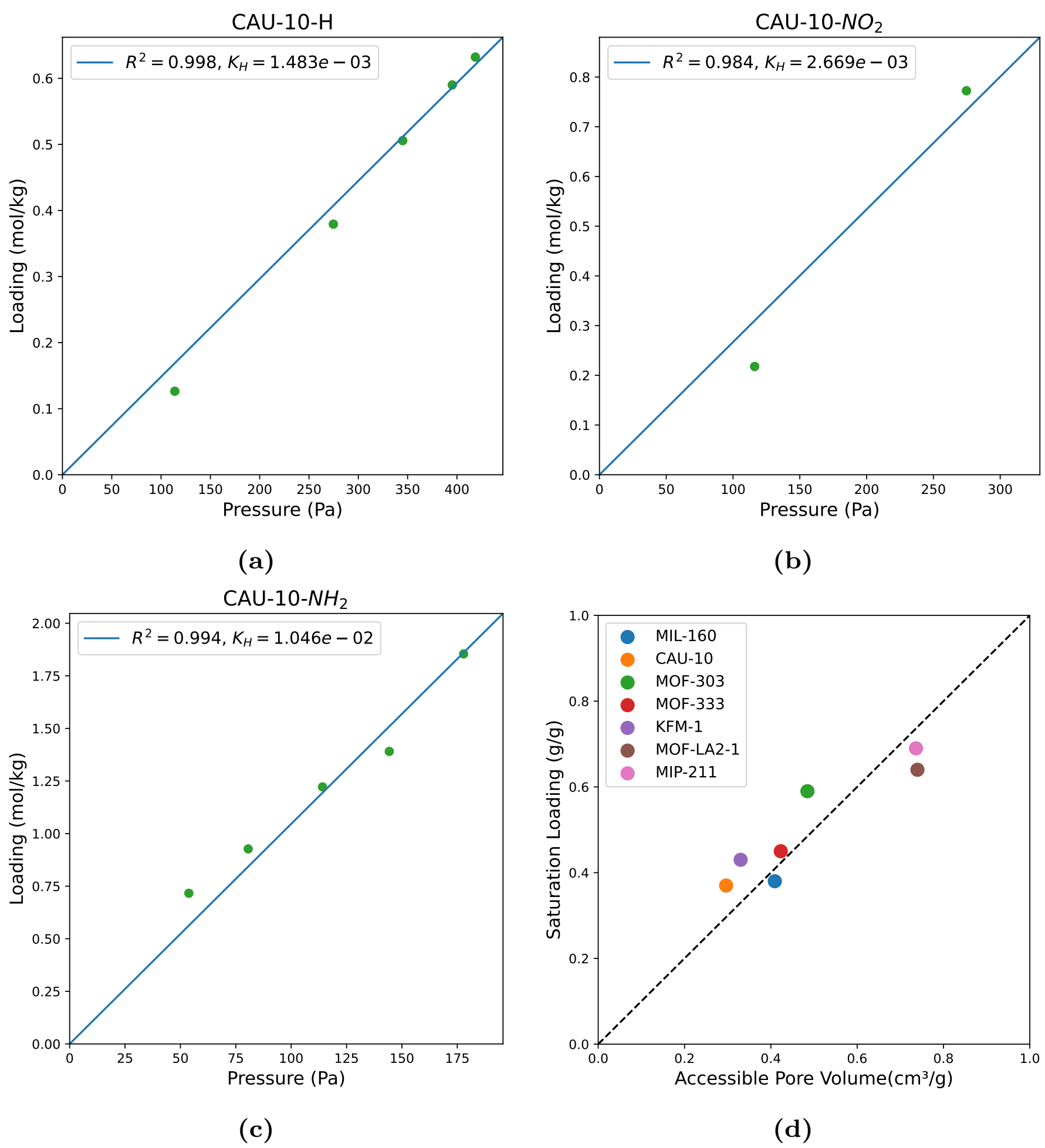

Figure S12: (a) Experimental water isotherm of CAU-10-H in the low-pressure region, where the slope is the Henry coefficient. (b) Experimental water isotherm of CAU-10-NO_2 in the low-pressure region, where the slope is the Henry coefficient. (c) Experimental water isotherm of CAU-10-NH_2 in the low-pressure region, where the slope is the Henry coefficient. (d) The experimental water uptakes in saturation conditions of 7 Al-MOFs as a function of the simulated accessible volume.[S14]

S5.2 Estimation of Saturation Water Uptakes

We can apply the liquid density ($\rho \approx 1.00\,\mathrm{g\,cm^{-3}}$) and the simulated accessible volume to estimate the saturated water uptakes. Here, for seven Al-MOFs, we plot the experimental saturated water uptakes[S14] as a function of the simulated accessible volume shown in Figure S12d. The good linear relationship shown in Figure S12d indicates that the simulated accessible volume is a good KPI for water uptake. From the screening studies, we have already searched optimal MOFs with similar $Q_{st}(H_2O)$ but significantly larger volumes.

S5.3 Calculation of Zero-loading Heat Adsorption

When we applied the fine-tuned model to simulate $Q_{\mathrm{st}}(H_2O)$ of MIP-211, we placed a single water molecule in the pristine MOF cell, which corresponds to a simulated loading of only 0.339,5 $\mathrm{mol\,kg^{-1}}$. In contrast, the $Q_{\mathrm{st}}(H_2O)$ values reported in the literature[S3] were measured at water uptakes of at least 4.4 $\mathrm{mol\,kg^{-1}}$, and are therefore not directly comparable with our simulation results. To enable a meaningful comparison, we followed the procedure described in Section S5.1 to extract $K_H(H_2O)$ from water adsorption isotherms at 288 K, 298 K, and 308 K. During this process, we note that the uptake of 0.339,5 $\mathrm{mol\,kg^{-1}}$ falls within the Henry regime (0 $\mathrm{mol\,kg^{-1}}$ to 4.0 $\mathrm{mol\,kg^{-1}}$ defined from water isotherms of MIP-211). We can apply these Henry coefficients to calculate the corresponding equilibrium pressures. As shown in Figure S13, the experimental $Q_{\mathrm{st}}(H_2O)$ derived from $K_H(H_2O)$ is 45.7 $\mathrm{kJ\,mol^{-1}}$. Because this $Q_{st}(H_2O)$ is actually derived from $K_H(H_2O)$, this value presents the $Q_{\mathrm{st}}(H_2O)$ at water uptakes within the Henry region, which is an ideal reference to compare with the simulated zero-loading $Q_{\mathrm{st}}(H_2O)$. This strategy also works for other MOFs.

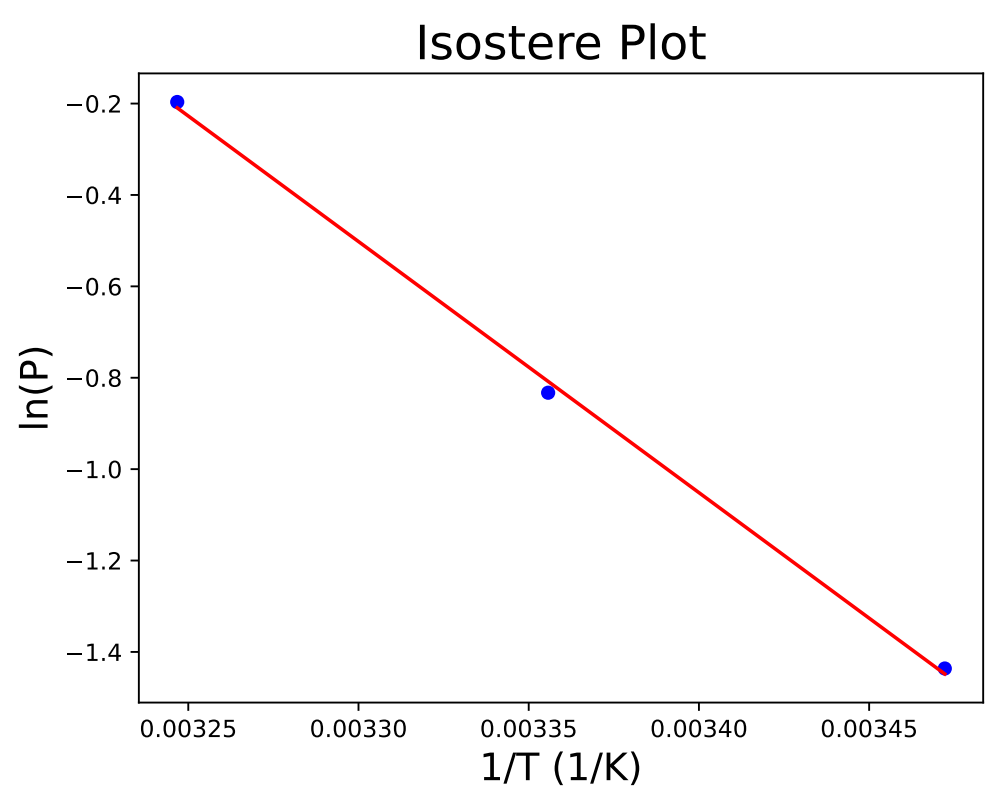

Figure S13: Water adsorption isosters for MIP-211, demonstrating the accuracy of $Q_{st}(H_2O)$ as evidenced by the linearity in the isoster

References

(S1) Deng, B.; Zhong, P.; Jun, K.; Riebesell, J.; Han, K.; Bartel, C. J.; Ceder, G. CHGNet as a pretrained universal neural network potential for charge-informed atomistic modelling. *Nature Machine Intelligence* **2023**, *5*, 1031–1041.

(S2) Batatia, I.; Benner, P.; Chiang, Y.; Elena, A. M.; Kovács, D. P.; Riebesell, J.; Advincula, X. R.; Asta, M.; Avaylon, M.; Baldwin, W. J.; others A foundation model for atomistic materials chemistry. *The Journal of chemical physics* **2025**, *163*.

(S3) Matemb Ma Ntep, T. J.; Wahiduzzaman, M.; Laurenz, E.; Cornu, I.; Mouchaham, G.; Dovgaliuk, I.; Nandi, S.; Knop, K.; Jansen, C.; Nouar, F.; others When polymorphism in metal–organic frameworks enables water sorption profile tunability for enhancing heat allocation and water harvesting performance. *Advanced Materials* **2024**, *36*, 2211302.

(S4) Cho, K. H.; Borges, D. D.; Lee, U.-H.; Lee, J. S.; Yoon, J. W.; Cho, S. J.; Park, J.; Lombardo, W.; Moon, D.; Sapienza, A.; others Rational design of a robust aluminum metal-organic framework for multi-purpose water-sorption-driven heat allocations. *Nature communications* **2020**, *11*, 5112.

(S5) Wang, F.; Ma, Z.; Cheng, J. Accelerating computation of acidity constants and redox potentials for aqueous organic redox flow batteries by machine learning potential-based molecular dynamics. *Journal of the American Chemical Society* **2024**, *146*, 14566–14575.

(S6) Cui, S.; Marandi, A.; Lebourleux, G.; Thimon, M.; Bourdon, M.; Chen, C.; Severino, M. I.; Steggles, V.; Nouar, F.; Serre, C. Heat properties of a hydrophilic carboxylate-based MOF for water adsorption applications. *Applied Thermal Engineering* **2019**, *161*, 114135.

(S7) Solovyeva, M.; Krivosheeva, I.; Gordeeva, L.; Aristov, Y. MIL-160 as an adsorbent for atmospheric water harvesting. *Energies* **2021**, *14*, 3586.

(S8) Zu, K.; Qin, M. Experimental and modeling investigation of water adsorption of hydrophilic carboxylate-based MOF for indoor moisture control. *Energy* **2021**, *228*, 120654.

(S9) Zhang, D.; Bi, H.; Dai, F.-Z.; Jiang, W.; Liu, X.; Zhang, L.; Wang, H. Pretraining of attention-based deep learning potential model for molecular simulation. *npj Computational Materials* **2024**, *10*, 94.

(S10) Sharma, A.; Sanvito, S. Quantum-accurate machine learning potentials for metal-organic frameworks using temperature driven active learning. *npj Computational Materials* **2024**, *10*, 237.

(S11) Vandenhaute, S.; Cools-Ceuppens, M.; DeKeyser, S.; Verstraelen, T.; Van Speybroeck, V. Machine learning potentials for metal-organic frameworks using an incremental learning approach. *npj Computational Materials* **2023**, *9*, 19.

(S12) Zhang, X.; Li, Y.; Jin, X.; Smit, B. FFLAME: a fragment-to-framework learning approach for MOF potentials. *Digital Discovery* **2025**, *4*, 3466–3477.

(S13) Solovyeva, M. V.; Shkatulov, A. I.; Gordeeva, L. G.; Fedorova, E. A.; Krieger, T. A.; Aristov, Y. I. Water vapor adsorption on CAU-10-X: effect of functional groups on adsorption equilibrium and mechanisms. *Langmuir* **2021**, *37*, 693–702.

(S14) Zhang, B.; Zhu, Z.; Wang, X.; Liu, X.; Kapteijn, F. Water adsorption in MOFs: structures and applications. *Advanced Functional Materials* **2024**, *34*, 2304788.
